# Comprehensively Profiling the Chromatin Architecture of Tissue Restricted Antigen Expression in Thymic Epithelial Cells Over Development

**DOI:** 10.3389/fimmu.2018.02120

**Published:** 2018-09-19

**Authors:** Adam E. Handel, Noriko Shikama-Dorn, Saule Zhanybekova, Stefano Maio, Annina N. Graedel, Saulius Zuklys, Chris P. Ponting, Georg A. Holländer

**Affiliations:** ^1^Nuffield Department of Clinical Neurosciences, University of Oxford, Oxford, United Kingdom; ^2^Department of Paediatrics, University of Oxford, Oxford United Kingdom; ^3^Department of Biomedicine, Universität Basel, Basel Switzerland; ^4^MRC Human Genetics Unit, University of Edinburgh, Edinburgh United Kingdom

**Keywords:** chromatin immunoprecipitation, histone modifications, thymic epithelial cells, AIRE, tissue restricted antigen

## Abstract

Thymic epithelial cells (TEC) effect crucial roles in thymopoiesis including the control of negative thymocyte selection. This process depends on their capacity to express promiscuously genes encoding tissue-restricted antigens. This competence is accomplished in medullary TEC (mTEC) in part by the presence of the transcriptional facilitator AutoImmune REgulator, AIRE. AIRE-regulated gene transcription is marked by repressive chromatin modifications, including H3K27me3. When during TEC development these chromatin marks are established, however, remains unclear. Here we use a comprehensive ChIP-seq dataset of multiple chromatin modifications in different TEC subtypes to demonstrate that the chromatin landscape is established early in TEC differentiation. Much of the chromatin architecture found in mature mTEC was found to be present already over earlier stages of mTEC lineage differentiation as well as in non-TEC tissues. This was reflected by the fact that a machine learning approach accurately classified genes as AIRE-induced or AIRE-independent both in immature and mature mTEC. Moreover, analysis of TEC specific enhancer elements identified candidate transcription factors likely to be important in mTEC development and function. Our findings indicate that the mature mTEC chromatin landscape is laid down early in mTEC differentiation, and that AIRE is not required for large-scale re-patterning of chromatin in mTEC.

## Introduction

Epithelial cells constitute the major stromal component of the thymus (1). These cells (designated thymic epithelial cells, TEC) form functionally and morphologically distinct anatomical regions, namely the outer cortex within which the early stages of T-cell development take place, and the inner medulla where the later stages of thymic T cell differentiation occur. One major developmental process in medullary TEC is progression from immature mTEC (marked by a lower cell surface expression of MHC class II molecules, and hence referred to as mTEC^lo^) to mature mTEC (phenotypically identified by high MHC class II expression, designated mTEC^hi^). TEC are critical for attracting blood-borne hematopoietic precursor cells and controlling their differentiation and selection to mature, functionally competent T cells via the sequential processes of positive and negative thymocyte selection (2). A key aspect of this selection process is the requirement for TEC as a population to express transcripts from virtually all protein-coding genes (3). This phenomenon is known as promiscuous gene expression (PGE) and requires the expression of the *Autoimmune Regulator* (*Aire*) gene, amongst others, to ensure transcription of around 4,000 tissue restricted antigens (TRA) within mTEC (3, 4). Mutations in *AIRE* result in the development of multi-system autoimmune disease in humans (5). Recently other genes, including *Fezf2, Prdm1*, and *Brg1*, have also been identified to regulate PGE (6–8).

The mechanism by which AIRE controls the expression of tissue specific genes is incompletely understood. The region around the transcriptional start site (TSS) of AIRE-regulated genes are more frequently marked by the repressive chromatin modification trimethylation of lysine-27 of histone H3 (H3K27me3) and less frequently by the promoter-associated H3K4me3 (3). When TEC are enriched for specific surface-expressed antigens, chromatin accessibility is greater around the TSS of those antigens than other genes (9). Patterns of tissue specific gene expression are known to occur independent of changes in DNA methylation (10). AIRE dynamically remodels chromatin to reduce chromatin accessibility and to tune the level of promiscuous gene expression across tissue specific genes (8).

The extent to which chromatin modifications in mTEC^lo^ and mTEC^hi^ determine AIRE regulatory status of tissue specific genes is poorly understood. In this study we demonstrate that much of the chromatin architecture observed in mTEC^hi^ is already present in mTEC^lo^ and construct computational models, based on the chromatin architecture around tissue specific genes, which accurately predict a gene's likelihood to be regulated by AIRE.

## Materials and methods

### Mice

C57BL/6 mice were obtained from Janvier (St. Berthevin, France). Mice were maintained under specific pathogen-free conditions. Experiments were in accordance with Swiss federal, cantonal, and institutional regulations.

### Isolation and sorting of thymic epithelial cells

Fragmented thymi were digested repeatedly for 15–20 min at 37°C with 1 unit/ml Liberase TM (Roche Diagnostic) and 100 μg/ml DNaseI (Roche Diagnostic) in PBS, to obtain single cell suspensions. After the final digest, cells were pooled and labeled with biotinylated anti-EpCAM for positive enrichment by AutoMACS system (Miltenyi Biotec), and stained using the following directly labeled antibodies and reagents: FITC-anti-IAb (clone AF6-120.1, BioLegend), PE-anti-Ly51 (clone 6C3, BioLegend), Alexa700-anti-CD45 (clone 30-F11, BioLegend), biotinylated anti-EpCAM (clone G8.8, DSHB, University of Iowa), PECy7-anti-Sca-1 (clone E13-161.7, Biolegend), Streptavidin-labeled PerCP-Cy5.5 (BioLegend), and Cy5-UEA1 (Vector Laboratories). The cells were exposed to 4′, 6-diamidino-2-phenylindole (DAPI) to identify dead cells and then sorted by flow cytometry (FACSAira II, BD Biosciences) achieving a TEC purity of over 93%. Sorted TEC were pelleted and cross-linked for ChIP and kept at −80°C until use.

### ChIP for histone markers

Chromatin immunoprecipitation (ChIP) was performed as previously described (3) using Protein A or G magnetic beads (Dynabeads, Life Technologies) to capture antibody-chromatin complexes. Antibodies used were anti-H3K4me1 (ab8895, Abcam), anti-H3K4me3 (C15410003, Diagenode), anti-H3K4ac (07-539, millipore), anti-H3K9ac (ab4441, Abcam), anti-H3K9me3 (05-1242, millipore), H3K27ac (ab4729, Abcam), and anti-H3K27me3 (07-449, millipore).

### Histone ChIP-seq analysis

We used FastQC to assess read quality and Trimmomatic to remove adapter sequences (transposons or their reverse complement), trim the first and last 3 bases of each read based on sequencing quality, trim sequences based on a sliding window (4:15), and retain reads with a minimum length of 20 bases (11). BWA (version 0.7.12) was used for pre-alignment of 100 base-pair paired-end reads against the UCSC mm10 genome with the arguments “bwa aln -t8 -q10 <forward/reverse reads>” and “bwa sampe <forward sai> <reverse sai>” (12). Pre-aligned bam files were further aligned with Stampy (version 1.0.23) with the arguments “-t 8 –process-part = n/10 –bamkeepgoodreads” (13). Reads were filtered to obtain concordantly mapping read pairs with a MAPQ score > 10. Picard Tools was used to remove duplicate fragments. Peaks for narrow peak marks (H3K4me1, H3K4ac, H3K9ac, and H3K27ac) were called using MACS2 (version 2.0.10.20131028) with the arguments “–keep-dup all” using pooled input samples as a control (14). Peaks were called for broad marks (H3K9me3 and H3K27me3) using MACS2 with the arguments “–keep-dup all –broad.” Peaks were filtered against the ENCODE mm10 blacklist (15, 16). Enrichment of ChIP-seq peaks within genes +/– 5 kb intervals was assessed using Genomic Association Tester (GAT) with 10,000 randomizations, using the appropriate sets of gene intervals as a workspace (17). Irreproducibility discovery rate were estimated for peaks as detailed in refs. (18, 19) using a threshold of IDR < 0.01. Pooled ChIP/input ratios were estimated for genes using the maximum signal within 1 kb of the TSS across all transcripts. Differential ChIP-seq peaks were identified using DiffBind (DBA_DESEQ2) with the default cut-off of FDR < 0.1 (20). Neural network modeled was undertaking using the package neuralnet in R. The optimum number of hidden nodes was estimated using iterative testing of fewer than 80% of the number of input nodes. A threshold of 0.01 improvement between iterations was used for neural network training on 67% of the total number of genes. The output threshold for gene categorization was chosen empirically based on the training set. The accuracy of the neural network was based on the correct categorization of the remaining 33% of genes. Null accuracy was defined as the accuracy of classification simply from resampling the test set categories. Contribution of different inputs to the neural network output was estimated using Olden's method, which estimates the contribution of each input variable to the neural network by summing the products of all hidden weights for each input and scaling this across all input variables (21).

### ATAC-seq

One replicate of ~10,000 wild-type cTEC, and two replicates each of ~25,000 mTEC^lo^ and mTEC^hi^ sorted in the same manner as described above underwent lysis, tagmentation, and PCR amplification as described in the ATAC-seq protocol (22). ATAC-seq libraries were sequenced on an Illumina HiSeq 2500.

### ATAC-seq analysis

We used FastQC to assess read quality and Trimmomatic to remove contaminating sequences (transpons or their reverse complements), crop the first and last 3 bases of each read based on sequencing quality, and remove the 3′ 10 bases of each read to remove partial transposon sequences (11). We used Bowtie2 (version 2.2.3) to align 100 base-pair paired-end reads against the UCSC mm10 genome with the arguments “–no-mixed –no-discordant -X 2000” as in a previous study (23). Reads were filtered to obtain concordantly mapping read pairs with a MAPQ score > 10. Picard Tools was used to remove duplicate fragments. The position of reads were passed into BEDtools and remapped taking into account transposon sequence insertion bias (24). Peaks were called using MACS2 (version 2.0.10.20131028) with the arguments “–nomodel –nolambda –keep-dup all –call-summits” as in a previous study (14, 25). Peaks were filtered against the ENCODE mm10 blacklist and a set of mitochondrial pseudopeaks generated from 1,000,0000 *in silico* 100 single-end reads produced from mitochondrial DNA aligned against non-mitochondrial DNA (15, 16).

### Single cell RNA-seq

mTEC^hi^ were isolated as detailed above and sorted into SMART-seq2 lysis buffer containing RNase inhibitors (26). Wells were spiked with 0.1 μl of 1:250,000 ERCC92 spike-in mix 1 (Ambion). Libraries were generated using the SMART-seq2 protocol and indexed using Nextera adapters before being sequenced on an Illumina HiSeq2500 platform.

### Single cell RNA-seq analysis

We used FastQC to assess read quality and Trimmomatic to remove contaminating sequences from reads then aligned these to the mm10 genome plus ERCC92 spike-ins using HISAT (version 0.1.6) 2-pass alignment (27). Gene quantitation was undertaken using HTSeq (with the option intersection non-empty) (28). Outlier cells were identified using robust PCA on alignment proportion, ERCC spike-in proportion, number of detectable genes, proportion of reads mapping to protein-coding genes, proportion of mitochondrial transcripts, proportion of ribosomal transcripts, 3′ to 5′ coverage bias, transcriptomic variance, cell-to-mean correlation, the proportion of the library accounted for by the top 500 transcripts and GC content (29). Counts were adjusted for library size using DESeq (30). FPKM values were converted to estimates of absolute molecule abundance using linear regression on ERCC92 spike-in expression. Matching of genes for AIRE status was undertaken by randomly matching AIRE induced genes with a tissue specific gene either similarly expressed in no cells or expressed in a very similar proportion of cells (within detection in one cell, i.e., ±0.6%). Genes for which there were no viable matches were discarded.

### Tissue specificity

The tissue specificity of genes was estimated using tau on the RNA-seq data available from the mouse ENCODE project (31, 32). *x*_*i*_ is the gene expression in tissue *i* where *n* is the number of tissues.

$$\tau =\frac{\sum_{i=1}^{n}(1-{\hat{x}}_{i})}{n-1};\;{\hat{x}}_{i}=\frac{x_{i}}{\begin{array}{c} \max(x_{i})\\ 1\;<\;i\;<\;n \end{array}}$$

### Data accessions

TEC histone ChIP-seq data has been deposited in GSE114713. mTEC^hi^ AIRE ChIP-seq data was downloaded from GSE92597. Additional RNA-seq and histone ChIP-seq data was obtained from the mouse ENCODE project (31, 32).

## Results

### Chromatin around AIRE-regulated genes is enriched for repressive marks and depleted in active marks

**Figure 1:**
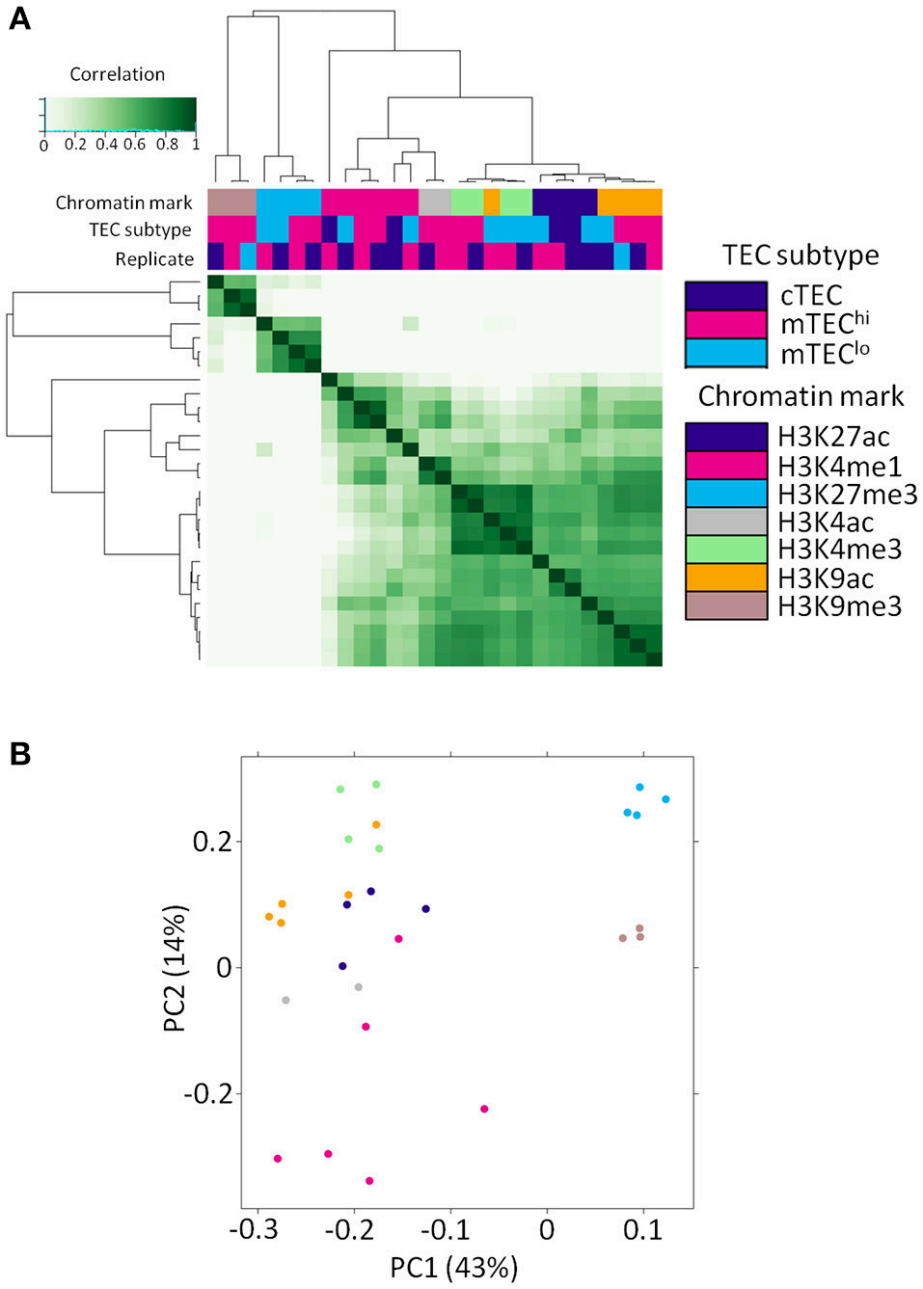
Histone ChIP-seq samples segregate primarily by chromatin mark. **(A)** Correlation heatmap of histone ChIP-seq samples. **(B)** Principal component analysis plot of histone ChIP-seq samples. The legend shows the color both for TEC subtype (for **A**) and chromatin mark (for **A,B**).

We generated replicated histone ChIP-seq data sets for distinct TEC subsets specific to each of multiple histone modifications (Supplementary Table 1). As expected, these samples clustered primarily by histone modification into repressive or activating marks on cross-correlation and principal component analysis (Figure 1). This comprehensive set of chromatin modifications allowed us to expand the number of chromatin modifications available for study around the TSS of genes regulated by AIRE in mTEC^hi^ and mTEC^lo^.

**Figure 2:**
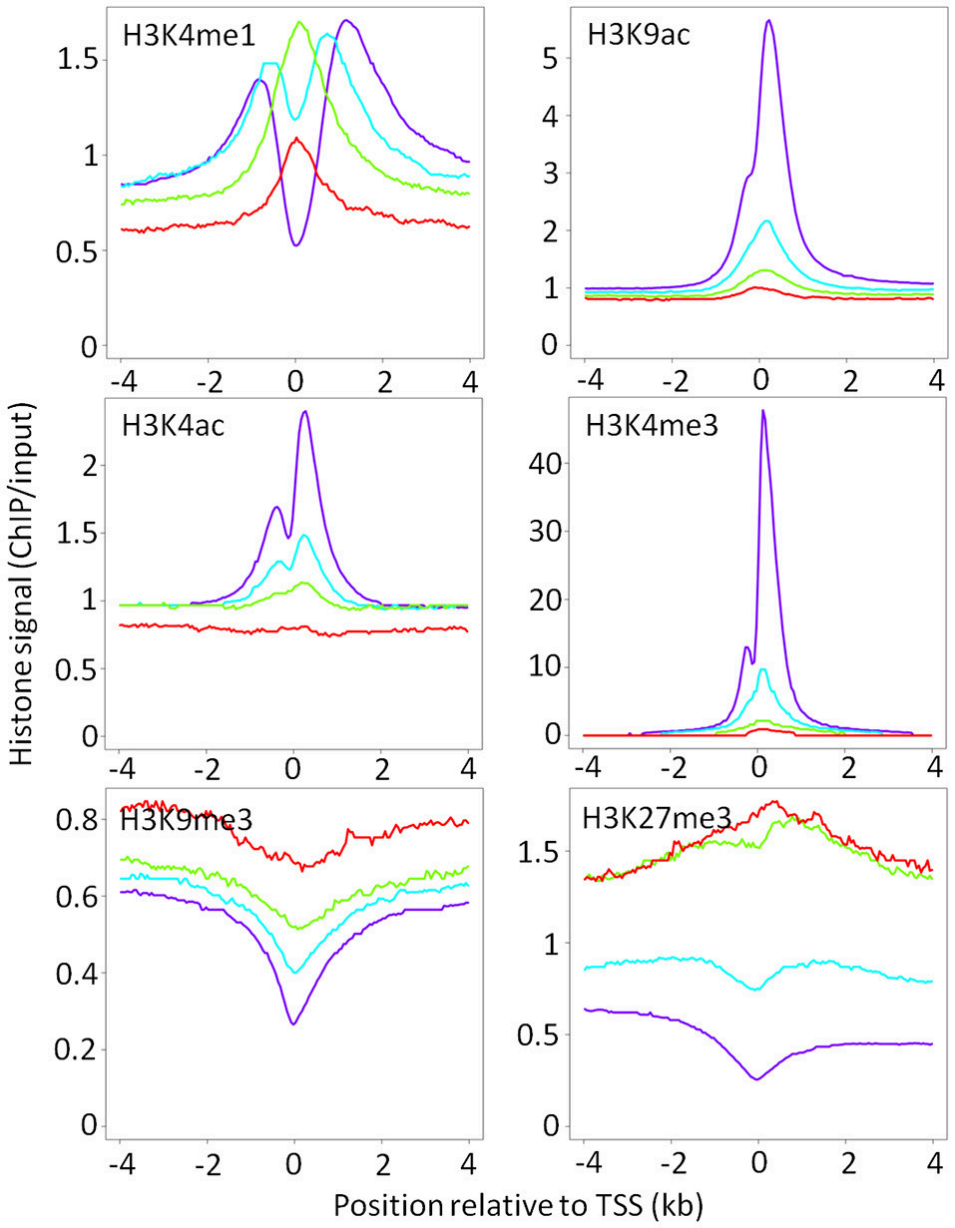
Chromatin landscape around the TSS of AIRE-induced and AIRE-independent genes in mTEC^hi^. Median ChIP/input signal scaled for library size is shown for each category of AIRE responsiveness (red = AIRE dependent; green = AIRE enhanced; blue = AIRE independent TRAs; purple = all other genes).

Genes were designated: as *AIRE-dependent*, if their transcripts were undetected in the absence of *Aire* expression; as *AIRE-enhanced*, if their expression was significantly increased >2-fold in the presence of AIRE relative to AIRE-negative mTEC; and, as *AIRE-independent*, if the presence of AIRE did not significantly change their expression in mTEC^hi^, a category which includes house-keeping genes. AIRE-independent genes were further divided into those with tissue restricted expression (TRAs) and those without tissue restricted expression (3). As previously reported by Sansom et al. AIRE dependent and enhanced genes showed elevated levels of the repressive chromatin modification, H3K27me3, around their TSS relative to AIRE-independent genes in mTEC^hi^, with the converse effect seen for the promoter-associated chromatin modification, H3K4me3 (Figure 2) (3). We further observed an elevation in a second repressive chromatin mark, H3K9me3, which was particularly pronounced around the TSS of AIRE-dependent genes. Enhancer-associated chromatin modifications, H3K4ac and H3K9ac, were reduced around the TSS of AIRE dependent and AIRE-enhanced genes relative to AIRE-independent genes. The distribution of H3K4me1 was altered around the TSS of both AIRE-dependent and -enhanced genes, with higher levels observed proximal to the TSS, whereas in AIRE independent genes H3K4me1 was marginalized to beyond 1 kb from the TSS. This pattern may suggest an ongoing process of H3K4me3 demethylation.

### Chromatin patterns in mTEC^hi^ and mTEC^lo^ are similar

**Figure 3:**
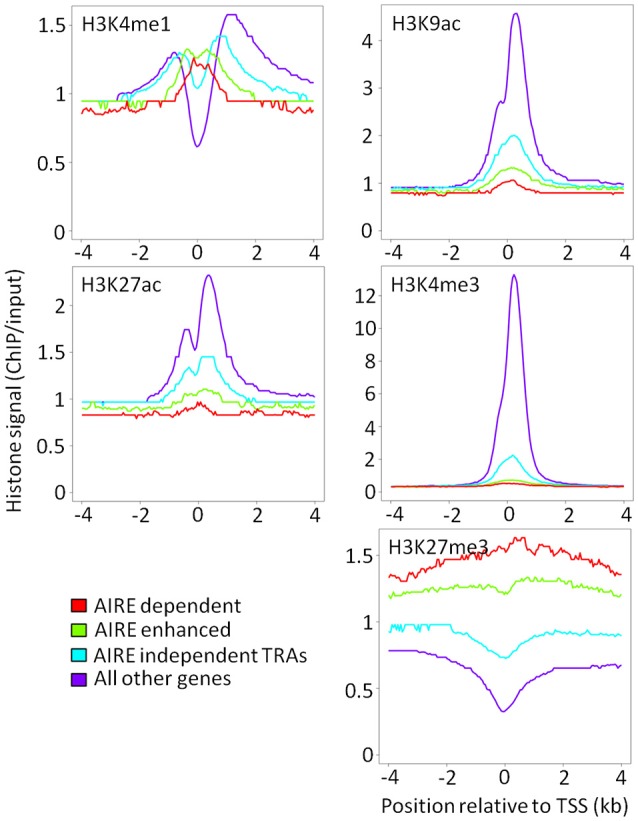
Chromatin landscape around the TSS of AIRE-induced and AIRE-independent genes in mTEC^lo^. Median ChIP/input signal scaled for library size is shown for each category of AIRE responsiveness (red = AIRE dependent; green = AIRE enhanced; blue = AIRE independent TRAs; purple = all other genes).

A key question in TEC promiscuous gene expression concerns the time point during mTEC lineage development when low levels of H3K4me3 and high levels of H3K27me3 marks are established, each a characteristic of AIRE regulated genes. We hypothesized that the higher proportional expression of TRAs observed in mTEC^hi^ than mTEC^lo^ would reflect differences in the underlying chromatin architecture between these mTEC subsets. Surprisingly, the overall pattern of chromatin modifications in mTEC^lo^ around AIRE-dependent, AIRE-enhanced or AIRE-independent genes was very similar to that observed in mTEC^hi^ (Figure 3). Despite this, it is possible that the magnitude of ChIP-seq peaks around AIRE-induced or AIRE-independent genes may differ between mTEC^lo^ and mTEC^hi^. In order to investigate this possibility, we identified differential histone ChIP-seq peaks between mTEC^lo^ and mTEC^hi^ using DiffBind (20). Enrichment of these mTEC subset-specific chromatin marks within the gene body and the flanking 5 kb of AIRE-induced or AIRE-independent genes was similar between mTEC^lo^ and mTEC^hi^ both for all genes and when restricting this analysis to tissue specific genes only (tissue specificity tau ≥0.8; Supplementary Figure 1). When we applied the same approach to high confidence histone ChIP-seq peaks (irreproducibility discovery rate [IDR] < 0.01) we again observed similar chromatin patterns in mTEC^lo^ and mTEC^hi^ (Supplementary Figures 2, 3). However, although the direction of ChIP-seq signal was similar between mTEC^lo^ and mTEC^hi^, active chromatin marks with significantly higher ChIP-seq signal in mTEC^hi^ showed a more extensive depletion around AIRE-induced genes (Supplementary Tables 2, 3). When analyzing the enrichment of all highly reproducible peaks around tissue restricted antigens between mTEC^hi^ and mTEC^lo^, the only significant difference observed was that H3K9ac depletion was more marked in mTEC^lo^ than mTEC^hi^ (Supplementary Tables 4, 5). Taken together, these results suggest that chromatin structure in mTEC^hi^ and mTEC^lo^ is broadly comparable.

It is possible that this similarity may reflect basal chromatin architecture present in non-TEC tissues. To explore this hypothesis, we used ENCODE histone ChIP-seq data to assess the same chromatin marks present in our mTEC^hi^ dataset around AIRE regulated or AIRE-independent genes. AIRE-dependent and AIRE-enhanced genes showed high levels of repressive chromatin marks and low levels of active chromatin marks in non-TEC tissues (Supplementary Figure 4). We hypothesized that this pattern may be driven by the level of gene expression in individual tissues. In order to investigate this, for each tissue we divided tissue specific genes into those maximally detected in that tissue and maximally detected in other tissues. This showed that individual tissue specific genes were characterized by high levels of active chromatin marks and low levels of repressive chromatin marks in tissues with high expression of those genes (Supplementary Figure 5). This suggests that the pattern of chromatin seen around AIRE responsive genes is present in multiple non-TEC tissues and is modulated by the tissue specific level of expression.

**Figure 4:**
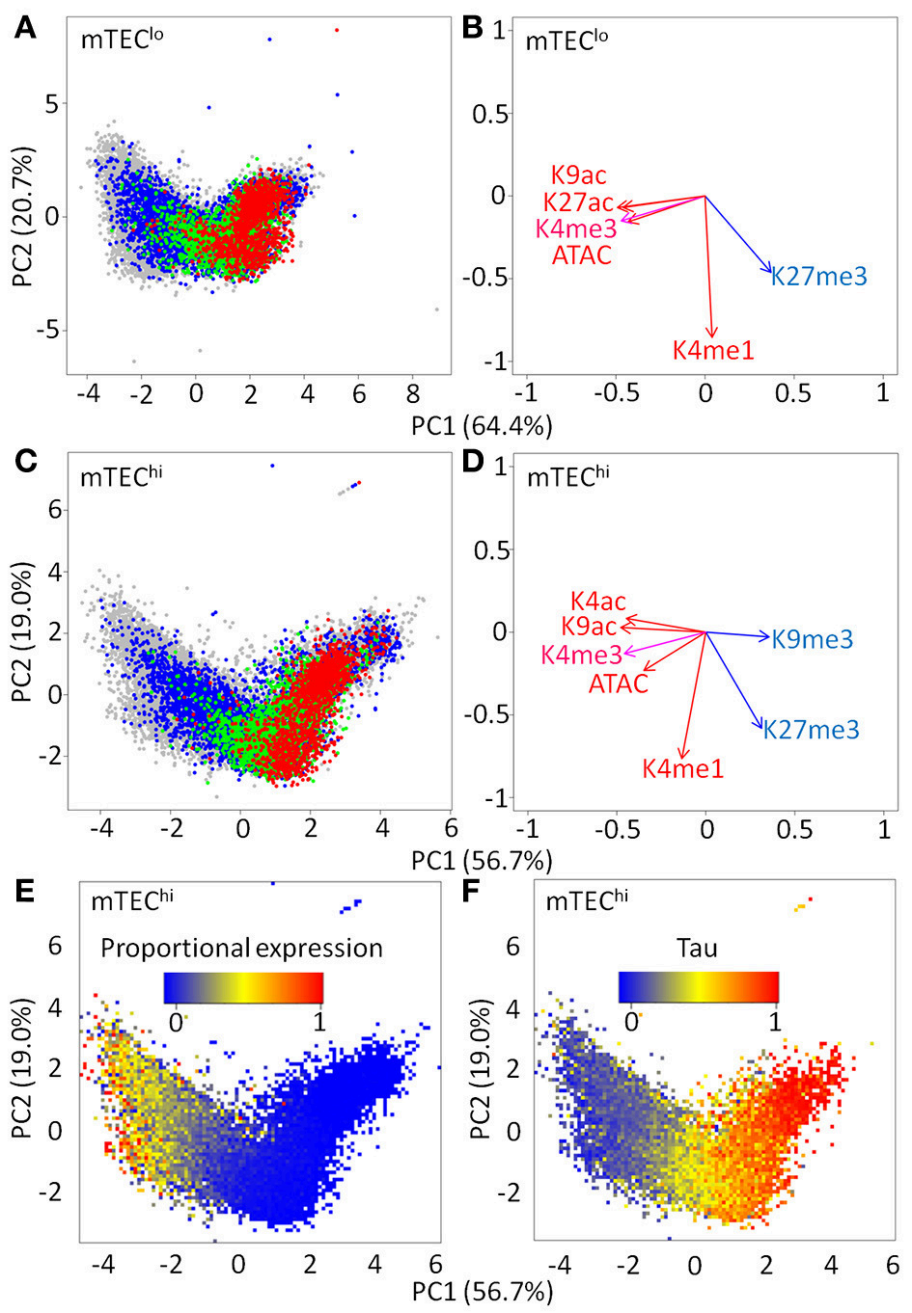
Principal component analysis (PCA) of chromatin signatures in mTEC^lo^ and mTEC^hi^. PCA plot of maximum signal within 1 kb of the TSS of individual genes [red = AIRE dependent; green = AIRE enhanced; blue = AIRE independent TRAs; gray = all other genes; **(A)** mTEClo and **(C)** mTEChi]. PCA rotations of individual chromatin marks for **(B)** mTEClo and **(D)** mTEChi. PCA heatmaps of genes shaded by the **(E)** proportion of single mTEC^hi^ expressing ≥ one molecule and **(F)** tau tissue specificity index.

We hypothesized that similarities in individual chromatin marks around TSS between mTEC^lo^ and mTEC^hi^ might persist when projected into higher dimensional space (Figures 4A–D; Supplementary Figures 6, 7). A clear distribution was present in either mTEC^lo^ or mTEC^hi^ that separated genes into those with high levels of repressive marks, preferentially regulated by AIRE, and those with high levels of activation marks that tended to be AIRE-independent. Given that AIRE-induced genes tend to be more lowly expressed than AIRE-independent genes, it was possible that this distribution could reflect underlying differences in the magnitude of gene expression. Indeed, proportional expression of genes in single mTEC^hi^ followed the same distribution as AIRE regulatory status (Figure 4E, Supplementary Figure 7; Spearman rho for PC1 *vs*. mTEC^hi^ proportional expression: rho = −0.81, *p* < 0.0001). Similar effects were seen for the magnitude of tissue specificity (Figure 4F; Spearman rho for PC1 vs. tissue specificity tau: rho = 0.73, *p* < 0.0001). Overall, this suggests that AIRE-dependent and AIRE-enhanced genes have a similar chromatin pattern in mTEC^hi^ and mTEC^lo^.

### Machine learning predicts AIRE responsiveness of genes from TSS chromatin contexts

**Figure 5:**
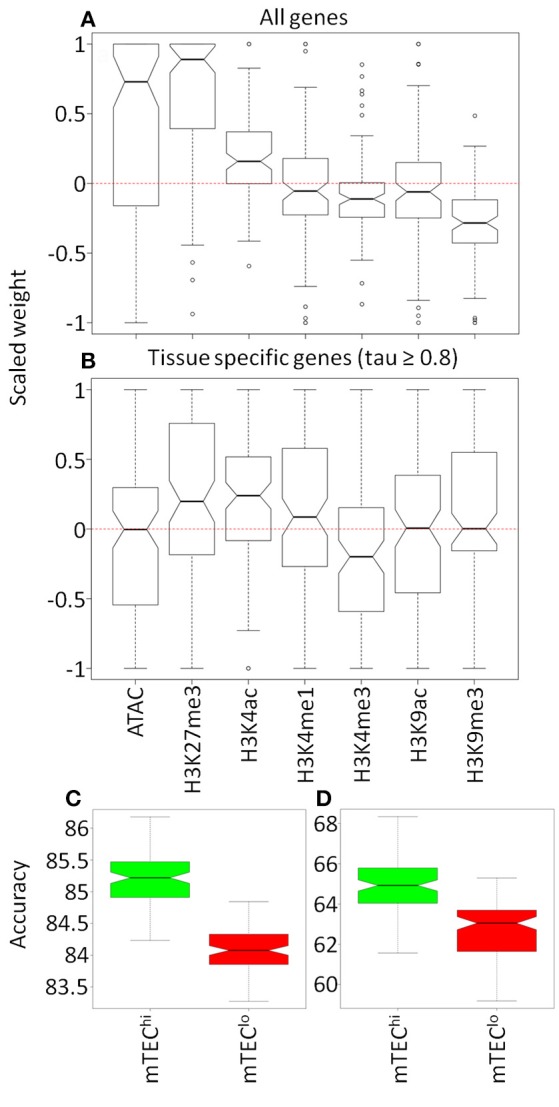
Comparison of models to classify genes by AIRE status. Olden weightings of mTEC^hi^ chromatin accessibility and histone modifications within 100 neural networks for **(A)** all genes and **(B)** tissue restricted genes (tau ≥ 0.8). Accuracy (%) of neural network models generated for histone marks in common between mTEC^lo^ (red) and mTEC^hi^ (green) for **(C)** all genes and **(D)** tissue restricted genes (tau ≥ 0.8).

The clear distribution of AIRE responsiveness in higher dimensional space encouraged us to assess whether machine learning methods could predict AIRE-induced or AIRE-independent status for genes based on the chromatin landscape surrounding genes' TSS. Neural networks were able to classify genes as AIRE-independent or AIRE-induced more accurately than expected by chance (mean accuracy: 85.3%; null accuracy: 69.9%; *p* < 0.0001; Supplementary Figure 8a). This remained accurate when the analysis was limited to high confidence TRAs (tau ≥ 0.8) (mean accuracy: 65.0%; null accuracy: 50.1%; *p* < 0.0001; Supplementary Figure 8b) or additionally to TRAs closely matched by proportional expression in single mTEC^hi^ (mean accuracy: 62.0%; null accuracy: 50.0%; *p* < 0.0001; Supplementary Figure 8c) (31). In the neural networks trained on all genes, chromatin accessibility, H3K27me3, and H3K4ac marks were associated with AIRE regulated genes whereas H3K4me3 and H3K9me3 modifications were associated with AIRE independence (*p* < 0.05; Figure 5A; Supplementary Figure 9a). When restricting the neural network analysis to only genes with tissue specific expression (tau ≥ 0.8), we found that only H3K27me3 and H3K4ac were associated with AIRE induced genes whereas H3K4me3 was associated with AIRE independent genes (*p* < 0.05; Figure 5B; Supplementary Figure 9b).

Limiting the neural network input to chromatin modifications available in both mTEC^hi^ and mTEC^lo^, we found that the accuracy of the model was better than by chance in either mTEC subtype (mean accuracy/null accuracy: all genes–mTEC^hi^ 85.2/69.9%, mTEC^lo^ 84.1/69.8%; tau ≥ 0.8–mTEC^hi^ 64.9/50.2%, mTEC^lo^ 62.8/50.0%; all *p* < 0.0001; Supplementary Figure 10). However, the accuracy of models derived from the chromatin architecture of mTEC^hi^ consistently outperformed those derived from mTEC^lo^ (*p* < 0.0001; Figures 5C,D). This increased accuracy from neural network modeling was associated with more consistent weighting given to specific chromatin modifications in mTEC^hi^ than mTEC^lo^, which may reflect a more consistent chromatin signature of AIRE responsiveness in mTEC^hi^ than mTEC^lo^ (Supplementary Figure 11).

### Chromatin marks around AIRE binding sites

Despite the chromatin architecture around TSS being similar in mTEC^lo^ and mTEC^hi^, it is possible that differences in chromatin marks at AIRE binding sites may underlie differential TRA expression in mTEC^lo^ and mTEC^hi^ (33). We found that AIRE binding sites were enriched for promoter and enhancer associated chromatin modifications and depleted in repressive chromatin marks relative to the remaining mappable genome (Supplementary Figure 12; Supplementary Table 6). Interestingly, there was no difference in the magnitude of this enrichment or depletion between mTEC^lo^ and mTEC^hi^ (*p* > 0.05), suggesting that differences in chromatin architecture at AIRE binding sites are unlikely to be the cause of transcriptomic differences between mTEC subtypes.

### Predicting transcription factor binding from enhancer chromatin modifications in TEC

**Figure 6:**
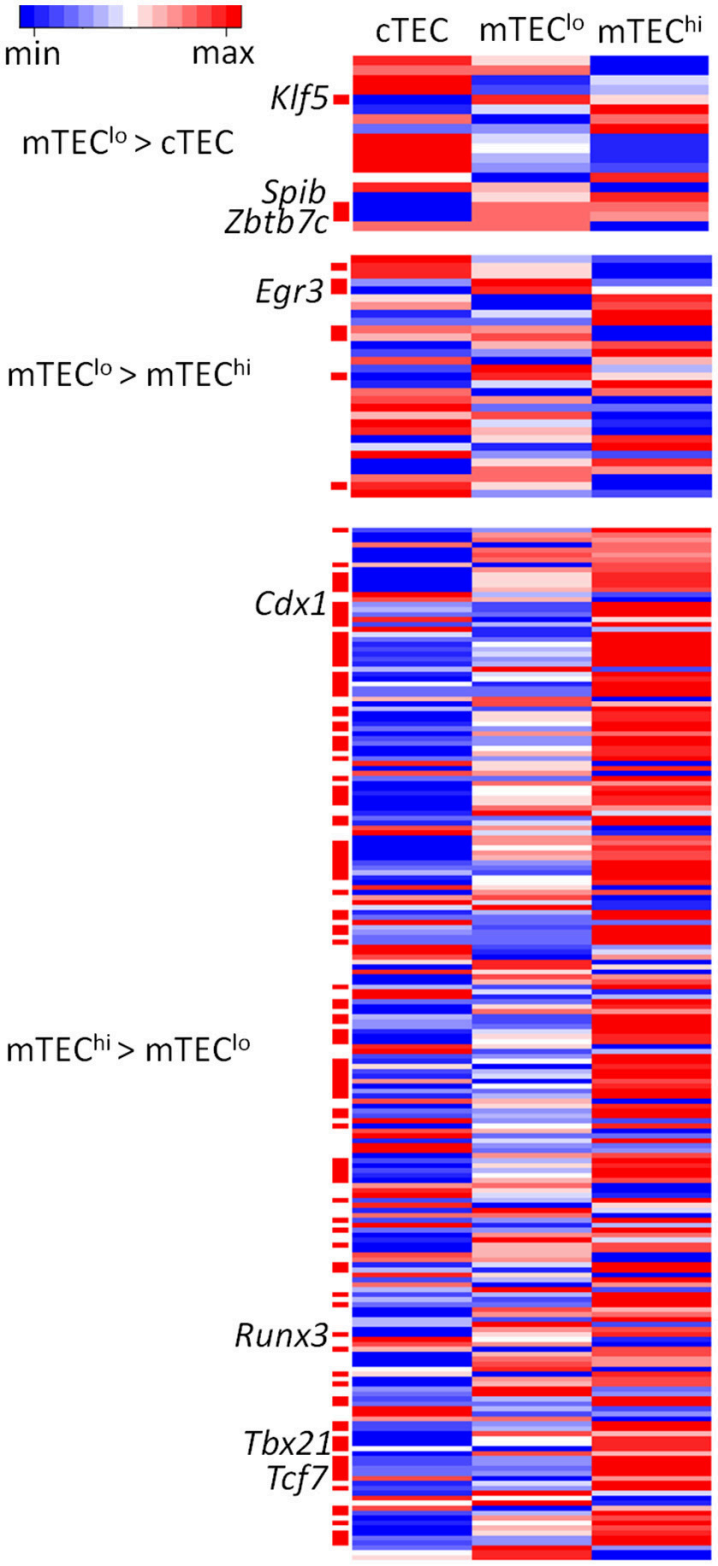
Heatmaps of RNA-seq data from TEC subtypes for significantly enriched transcription factor motifs within enhancer elements. Transcription factor motifs differentially expressed between the TEC subtypes shown are indicated by the red bars. Transcription factors expressed at FPKM > 10 and with a fold change > 5 are indicated by text to the left of the heatmap. Expression values are log_2_ transformed and scaled by gene.

Beyond AIRE, the binding of transcription factors may shape specific differences between mTEC^lo^ and mTEC^hi^. We therefore assessed the enrichment of transcription factor binding motifs curated from JASPAR, the open access data base of non-redundant transcription factor binding sites, to assess the enrichment of motifs within peaks containing enhancer chromatin modifications differentially identified between TEC subtypes (Supplementary Figures 13, 14). By intersecting enriched motifs between different enhancer marks and overlaying this motif enrichment on transcriptomic data, we found candidate transcription factors with motifs that were differentially enriched within enhancers and differentially expressed between relevant TEC subtypes (FDR < 0.05; Figure 6; Supplementary Table 7). We identified candidate transcription factors particularly likely to be important for the differentiation or function of specific TEC subtypes by highlighting motifs expressed at FPKM > 10 and with a fold change > 5 between TEC subtypes. This approach identified: *Klf5, Spib*, and *Zbtb7c* in mTEC^lo^ > cTEC, *Egr3* in mTEC^lo^ > mTEC^hi^, and *Cdx1, Runx3, Tbx21*, and *Tcf7* in mTEC^hi^ > mTEC^lo^.

### Gene ontology analysis of chromatin marks

Even without large-scale alterations in chromatin patterns, it is likely that differences in chromatin marks between TEC subtypes may be enriched near genes involved with particular biological and molecular processes. An overlap analysis using each of the available chromatin marks in cTEC, mTEC^lo^ and mTEC^hi^ revealed enrichment in multiple different biological pathways (Supplementary Figure 15). As above, we identified chromatin marks with significantly higher ChIP-seq signal in specific TEC subsets. Based on our motif analysis, one molecular pathway of particular interest in mTEC^lo^ chromatin modifications was the enrichment of H3K4me1 and H3K27ac peaks within sets of genes known to be upregulated by epithelial growth factor (EGF) (H3K4me1 1.9-fold, *q* < 10^−7^; H3K27ac 3.03-fold, *q* < 10^−11^). Enhancer marks specifically present in mTEC^hi^ were enriched for a multitude of mouse phenotypes associated with abnormal lymphocyte development and function (e.g., abnormal CD4+ T-cell physiology: H3K4me1 2.0-fold, *q* < 10^−8^; H3K9ac 2.2-fold, *q* < 10^−12^) as well as gene pathways upregulated in response to ionizing radiation (H3K4me1 2.05-fold, *q* < 10^−6^; H3K9ac 2.3-fold, *q* < 10^−9^). H3K27me3 peaks in mTEC^lo^, but notably not in mTEC^hi^, were enriched for known targets of the Polycomb Repressive Complex 2 (mTEC^lo^: 2.8-fold, *q* < 10^−17^; no overlapping genes in mTEC^hi^).

## Discussion

We have identified differences in chromatin architecture between AIRE-regulated and AIRE-independent genes. Dimensionality reduction of the observed histone modifications revealed a clear separation of genes by AIRE regulatory status in mTEC. This distribution was also associated with tissue specificity and proportional expression in mTEC^hi^. Machine learning through neural network analysis was able to predict the AIRE status of genes from multidimensional measures of chromatin architecture in both mTEC^lo^ and mTEC^hi^, although with significantly higher accuracy in mature over immature mTEC. Together these findings suggested that the chromatin architecture is broadly similar between mTEC^lo^ and mTEC^hi^ but is further refined through the course of mTEC differentiation with a more marked reduction in most active chromatin marks around AIRE-induced genes in mTEC^hi^ than in mTEC^lo^. Moreover, an analysis of chromatin modifications was also able to identify potential novel master transcription factors of TEC development and functional pathways in which chromatin modifications specific to TEC subtypes were significantly enriched.

Our data suggest that much of the chromatin landscape surrounding tissue specific genes is already present in mTEC^lo^ prior to mTEC^hi^ differentiation. Previous studies only examined histone modifications in mTEC^hi^. Consequently, the chromatin landscape prior to this point in TEC differentiation was previously unknown (3, 33). However, supportive evidence that this might be expected to be the case was provided by the observation, which we have expanded upon in this study, that the chromatin patterns around AIRE-induced genes in mouse ENCODE ChIP-seq data derived from non-TEC cell types are similar to those observed in mTEC^hi^ (3). This suggests that AIRE is not required to establish the chromatin architecture of tissue specific antigens but instead acts dynamically to ensure appropriate levels of histone modifications, as suggested by a previous chromatin *in vivo* assay (8). Our machine learning approaches support the fact that AIRE status can be predicted from chromatin signatures in both mTEC^lo^ and mTEC^hi^. One important caveat to these findings is that a small proportion of mTEC^lo^ cells are actually terminally differentiated post-AIRE mTEC (34). Although this could dilute the magnitude of any differential signal between mTEC^lo^ and mTEC^hi^, the relatively small size of this population of post-AIRE cells is unlikely to have a major impact on our analysis. In other systems, reshaping of the chromatin architecture occurs *after* alterations in transcription and it is possible that chromatin patterning in mTEC^hi^ is determined *by* transcription rather than transcription being determined by chromatin marks (35). Knock-out of determinants of epigenomic remodeling will be required to resolve this issue.

The key transcription factor motifs identified in enhancer elements within specific TEC subsets highlighted potential master regulators of TEC development and function, each of which was robustly expressed (FPKM > 10) in TEC. *Klf5, Runx3, Spib*, and *Zbtb7c* are known to regulate thymocyte development but have not been studied in TEC (36–38). *Egr3* is involved in γδ T-cell development (39). *Tbx21* and *Tcf7* have previously been implicated in the expression of AIRE in mTEC (40). Of particular interest are the transcription factors that differ between cTEC and mTEC^lo^ both in enhancer availability and transcript expression (*Klf5, Spib*, and *Zbtb7c*), as these may be instrumental in driving the bifurcation between cTEC and mTEC fate from the early, bipotent progenitor stage onwards (41). Further work should focus on the functional effects of these transcription factors on TEC progenitors.

Our gene ontology analysis (Supplementary Figure 15) of multiple different chromatin marks identified pathways involved in immune system function. Our finding that there was additionally enrichment of pathways involved in the response to ionizing radiation in mTEC^hi^ was interesting because AIRE is thought to cause DNA double-strand breaks as part of its dynamic remodeling of chromatin (42). It was also noteworthy that H3K27me3 peaks in mTEC^lo^ were enriched for genes known to be conventional targets of the Polycomb Repressive Complex 2 but this was not the case in mTEC^hi^. This suggests that dynamic remodeling of repressive chromatin marks may differ over the course of mTEC maturation.

An important limitation of the approaches currently applied to study the chromatin architecture of TEC is that requirements for large cell numbers mean that histone modifications represent a population average as these can only practically be surveyed on pooled cells. Studies in which mTEC^hi^ have been purified for cells expressing specific tissue specific genes revealed that their chromatin accessibility and that of co-expressed genes were substantially higher relative to other loci (9). Given the stochastic expression of genes in individual mTEC^hi^, this observation suggests that population level measures of chromatin modifications are unlikely to capture the state of individual cells (3, 9, 10). The future application of single cell techniques that permit the parallel measurement of the transcriptome and chromatin accessibility will help to clarify the chromatin landscape in individual mTEC and correlate their state to the expression of particular tissue specific genes (43).

## Ethics statement

This study was carried out in accordance with the recommendations of local guidelines, Kantonales Veterinäramt BS. The protocol was approved by the Kantonales Veterinäramt BS.

## Author contributions

AH, NS-D, SZh, SM, and GH designed the experiments. NS-D, SZh, and SM performed the experiments. AH analyzed the data. AH, CP, and GH wrote the manuscript. All authors critically revised the manuscript.

### Conflict of interest statement

The authors declare that the research was conducted in the absence of any commercial or financial relationships that could be construed as a potential conflict of interest.

## Supplementary material

Supplementary Figure 1 Enrichment of differential histone ChIP-seq peaks within genes of different AIRE status. GAT was used to test enrichment of histone ChIP-seq peaks with differential signal in mTEC^lo^ or mTEC^hi^ within AIRE independent (blue) and AIRE induced (red) genes +/– 5 kb. Error bars indicate 95% confidence intervals from 10,000 permutations. Enrichment is relative to all genes (top) or tissue specific genes (tau ≥ 0.8).

Supplementary Figure 2 Enrichment of mTEC^lo^ histone ChIP-seq peaks (IDR < 0.01) within genes of different AIRE status. GAT was used to test enrichment of histone ChIP-seq peaks (IDR < 0.01) in mTEC^lo^ within AIRE independent (blue) and AIRE induced (red) genes +/– 5 kb. Error bars indicate 95% confidence intervals from 10,000 permutations. Enrichment is relative to all genes (top) or tissue specific genes (tau ≥ 0.8).

Supplementary Figure 3 Enrichment of mTEC^hi^ histone ChIP-seq peaks (IDR < 0.01) within genes of different AIRE status. GAT was used to test enrichment of histone ChIP-seq peaks (IDR < 0.01) in mTEC^hi^ within AIRE independent (blue) and AIRE induced (red) genes +/– 5 kb. Error bars indicate 95% confidence intervals from 10,000 permutations. Enrichment is relative to all genes (top) or tissue specific genes (tau ≥ 0.8).

Supplementary Figure 4 Chromatin signals in ENCODE tissues. Boxplots show log_2_ ChIP/input ratio scaled by library size within 1 kb of the TSS of genes that are AIRE-dependent, AIRE-enhanced, AIRE-independent TRAs or all other genes. Kruskal-Wallis *p* < 0.0001 for all tissues.

Supplementary Figure 5 Chromatin signals in ENCODE tissues near tissue specific genes. Boxplots show log_2_ ChIP/input ratio scaled by library size within 1 kb of the TSS of genes with tissue specificity tau ≥ 0.8. Genes are divided into AIRE-induced and AIRE-independent, and additionally for each tissue into genes maximally expressed in each tissue or not maximally expressed in that tissue. Kruskal-Wallis *p* < 0.05 for all tissues.

Supplementary Figure 6 Heatmaps of chromatin signals in mTEC^lo^. ATAC-seq signal is expressed as log_2_ CPM+1. ChIP-seq signal is expressed as log_2_ ChIP/input ratio scaled by library size. The bottom panel shows tau tissue specificity index.

Supplementary Figure 7 Heatmaps of chromatin signals in mTEC^hi^. ATAC-seq signal is expressed as log_2_ CPM+1. ChIP-seq signal is expressed as log_2_ ChIP/input ratio scaled by library size.

Supplementary Figure 8 Plots of accuracy of neural networks compared with null accuracy. **(a)** All genes, **(b)** tissue restricted genes (tissue specificity tau ≥ 0.8), and **(c)** tissue restricted genes (tissue specificity tau ≥ 0.8) closely matched on proportional expression in single mTEC^hi^. The null accuracy was estimated by randomly sampling the true gene categories for each 100 neural networks.

Supplementary Figure 9 Optimum neural networks for AIRE categorization. Plots of the neural networks are shown for the network with the best accuracy for **(a)** all genes and **(b)** tissue restricted genes (tau ≥ 0.8).

Supplementary Figure 10 Accuracy of neural networks for predicting AIRE status of genes.

Supplementary Figure 11 Olden weighting of chromatin accessibility and histone modifications in neural networks for predicting AIRE status of genes.

Supplementary Figure 12 Enrichment of chromatin modifications within AIRE binding sites. GAT was used to test enrichment of histone ChIP-seq peaks (IDR < 0.01) in mTEC^lo^ (red) and mTEC^hi^ (green) within AIRE ChIP-seq peaks (IDR < 0.01) relative to the rest of the mappable genome. Error bars indicate 95% confidence intervals from 10,000 permutations.

Supplementary Figure 13 Significantly enriched JASPAR motifs in cTEC (blue) or mTEC^lo^ (red) for H3K4me1 (top) or H3K27ac (bottom). The top 20 significant motifs (FDR < 0.05) are shown ordered by enrichment.

Supplementary Figure 14 Significantly enriched JASPAR motifs in mTEC^lo^ (red) or mTEC^hi^ (green) for H3K4me1 (top) or H3K9ac (bottom). The top 20 significant motifs (FDR < 0.05) are shown ordered by enrichment.

Supplementary Figure 15 (left) Gene ontology enrichment from GREAT on differential peaks in cTEC (blue) or mTEC^lo^ (red); (right) Gene ontology enrichment from GREAT on differential peaks in mTEC^lo^ (red) or mTEC^hi^ (green). The top 20 significant terms (FDR < 0.05) ranked by fold enrichment are shown. Only categories with greater than 50 and fewer than 1,000 genes are shown.

Supplementary Table 1 Experimental design. ^*^ = samples generated as part of a previous study (3).

Supplementary Table 2 Enrichment of histone ChIP-seq peaks differentially detected in mTEC^lo^ or mTEC^hi^ within AIRE-enhanced and AIRE-independent genes. GAT was used to test enrichment of histone ChIP-seq peaks with differential signal in mTEC^lo^ or mTEC^hi^ within AIRE independent (blue) and AIRE induced (red) genes +/– 5 kb.

Supplementary Table 3 Enrichment of histone ChIP-seq peaks differentially detected in mTEC^lo^ or mTEC^hi^ within AIRE-enhanced and AIRE-independent TRAs. GAT was used to test enrichment of histone ChIP-seq peaks with differential signal in mTEC^lo^ or mTEC^hi^ within AIRE independent (blue) and AIRE induced (red) TRAs +/– 5 kb.

Supplementary Table 4 Enrichment of histone ChIP-seq peaks detected in mTEC^lo^ or mTEC^hi^ (IDR < 0.01) within AIRE-enhanced and AIRE-independent genes. GAT was used to test enrichment of histone ChIP-seq peaks within AIRE independent (blue) and AIRE induced (red) genes +/– 5 kb.

Supplementary Table 5 Enrichment of histone ChIP-seq peaks detected in mTEC^lo^ or mTEC^hi^ (IDR < 0.01) within AIRE-enhanced and AIRE-independent TRAs. GAT was used to test enrichment of histone ChIP-seq peaks within AIRE independent (blue) and AIRE induced (red) TRAs +/– 5 kb.

Supplementary Table 6 Enrichment of histone ChIP-seq peaks within AIRE ChIP-seq peaks.

Supplementary Table 7 Motif enrichment within different TEC subtypes.
