# Supplementary figures and images for: Comprehensively Profiling the Chromatin Architecture of Tissue Restricted Antigen Expression in Thymic Epithelial Cells Over Development
Source: Front Immunol. 2018 Sep 19;9:2120. doi: 10.3389/fimmu.2018.02120 (PMC6156148; doi:10.3389/fimmu.2018.02120)

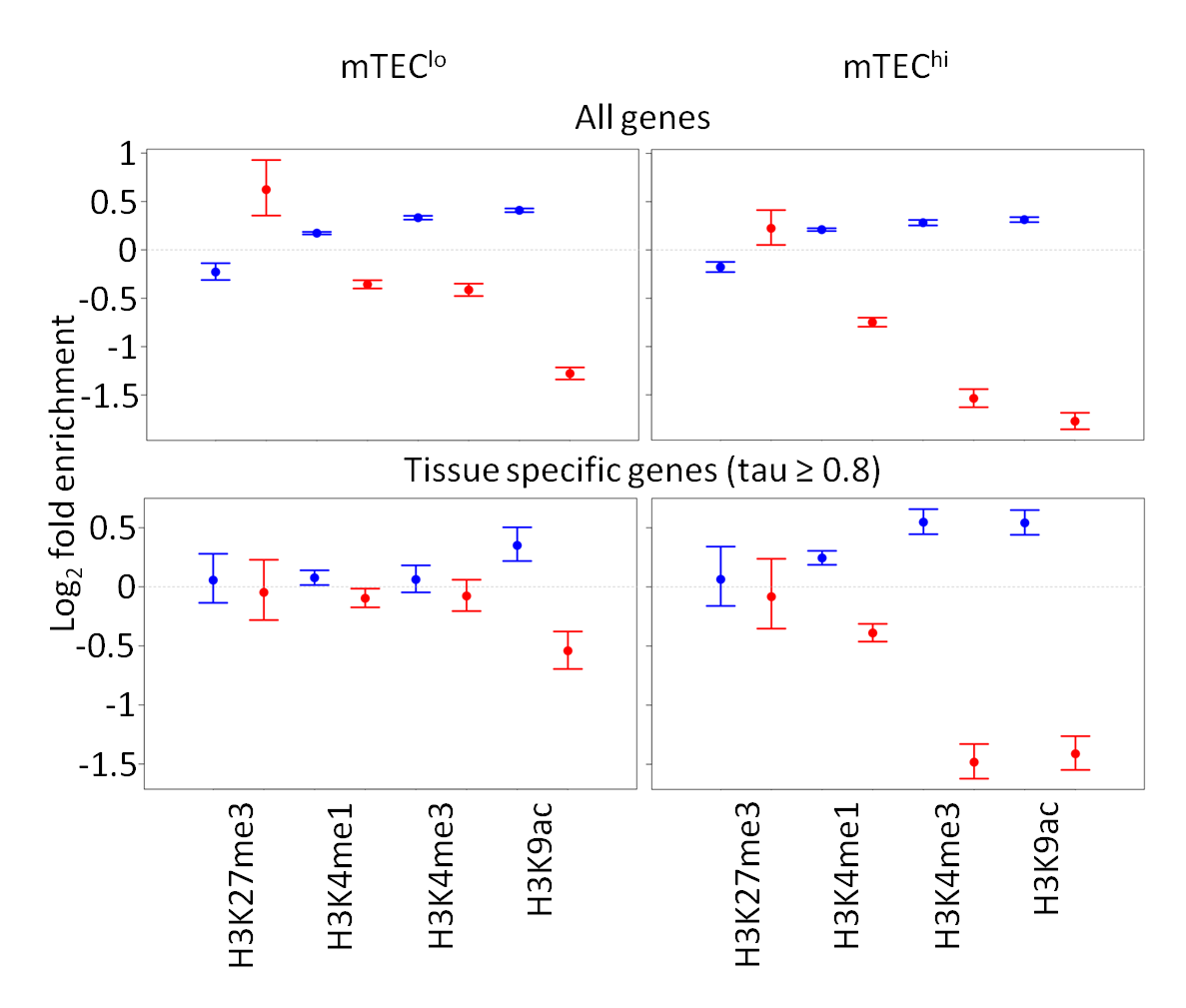

Supplement: Supplementary Figure 1 — Enrichment of differential histone ChIP-seq peaks within genes of different AIRE status. GAT was used to test enrichment of histone ChIP-seq peaks with differential signal in mTEClo or mTEChi within AIRE independent (blue) and AIRE induced (red) genes +/– 5 kb. Error bars indicate 95% confidence intervals from 10,000 permutations. Enrichment is relative to all genes (top) or tissue specific genes (tau ≥ 0.8). [file Image_1.PNG]

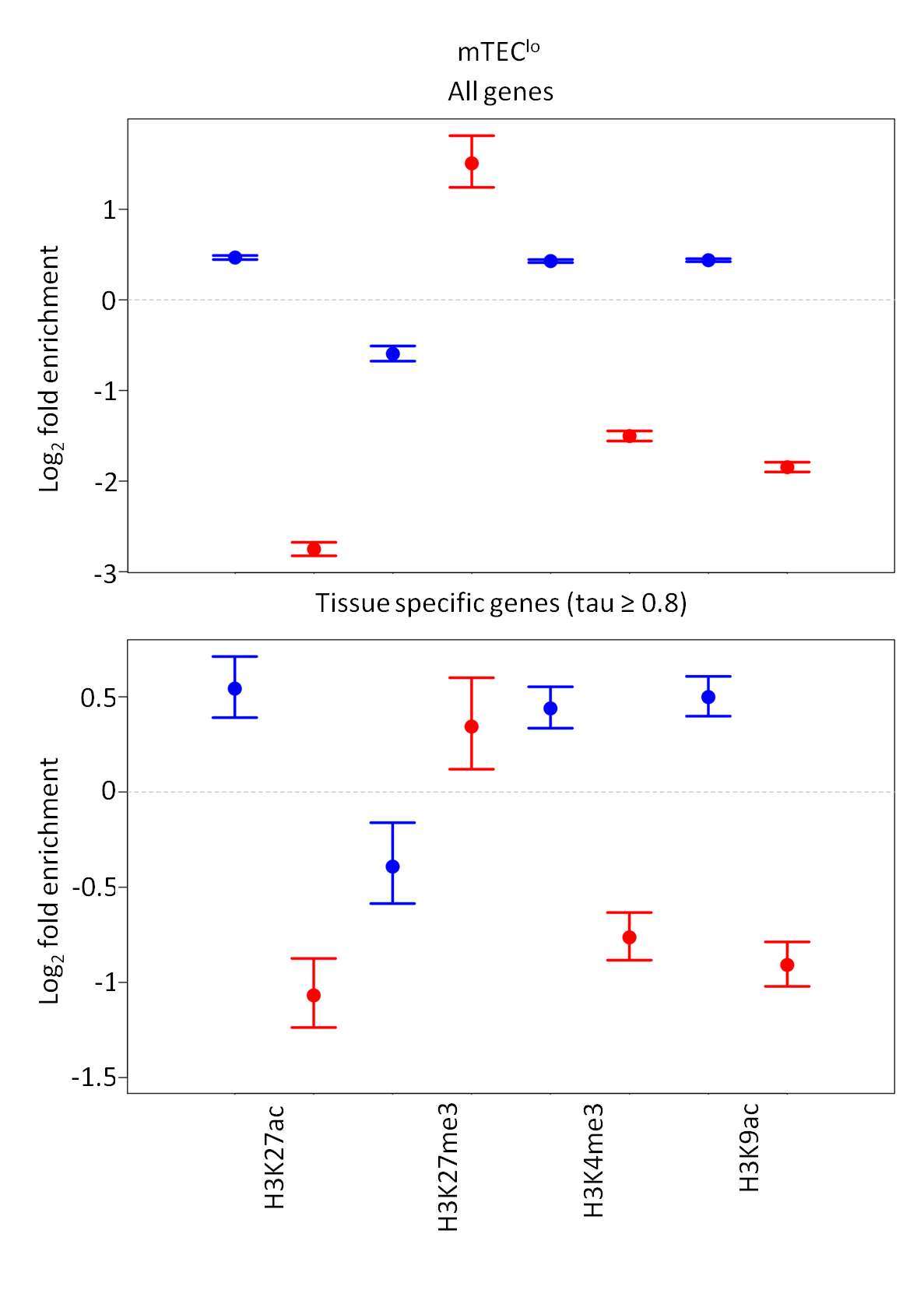

Supplement: Supplementary Figure 2 — Enrichment of mTEClo histone ChIP-seq peaks (IDR < 0.01) within genes of different AIRE status. GAT was used to test enrichment of histone ChIP-seq peaks (IDR < 0.01) in mTEClo within AIRE independent (blue) and AIRE induced (red) genes +/– 5 kb. Error bars indicate 95% confidence intervals from 10,000 permutations. Enrichment is relative to all genes (top) or tissue specific genes (tau ≥ 0.8). [file Image_2.PNG]

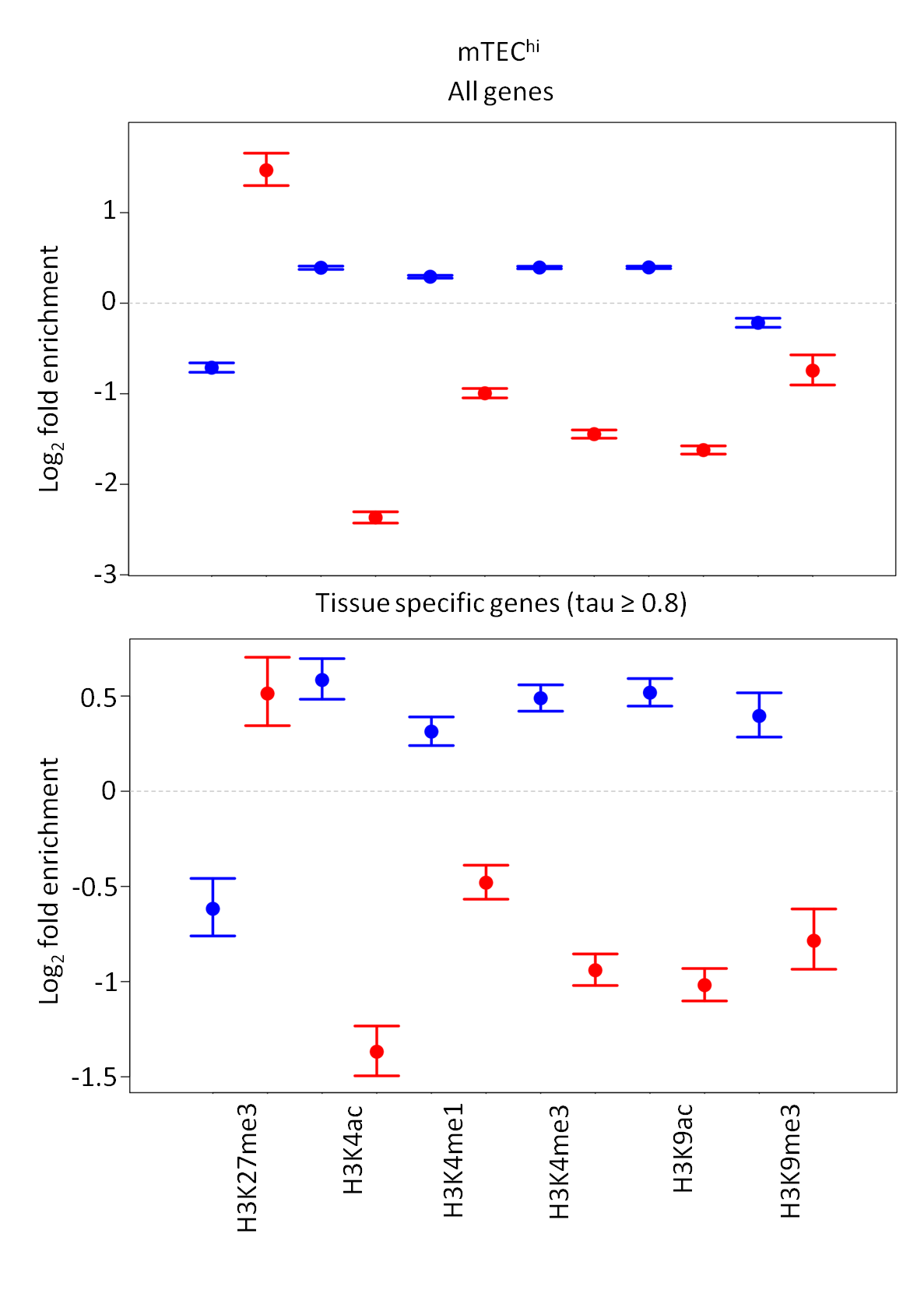

Supplement: Supplementary Figure 3 — Enrichment of mTEChi histone ChIP-seq peaks (IDR < 0.01) within genes of different AIRE status. GAT was used to test enrichment of histone ChIP-seq peaks (IDR < 0.01) in mTEChi within AIRE independent (blue) and AIRE induced (red) genes +/– 5 kb. Error bars indicate 95% confidence intervals from 10,000 permutations. Enrichment is relative to all genes (top) or tissue specific genes (tau ≥ 0.8). [file Image_3.PNG]

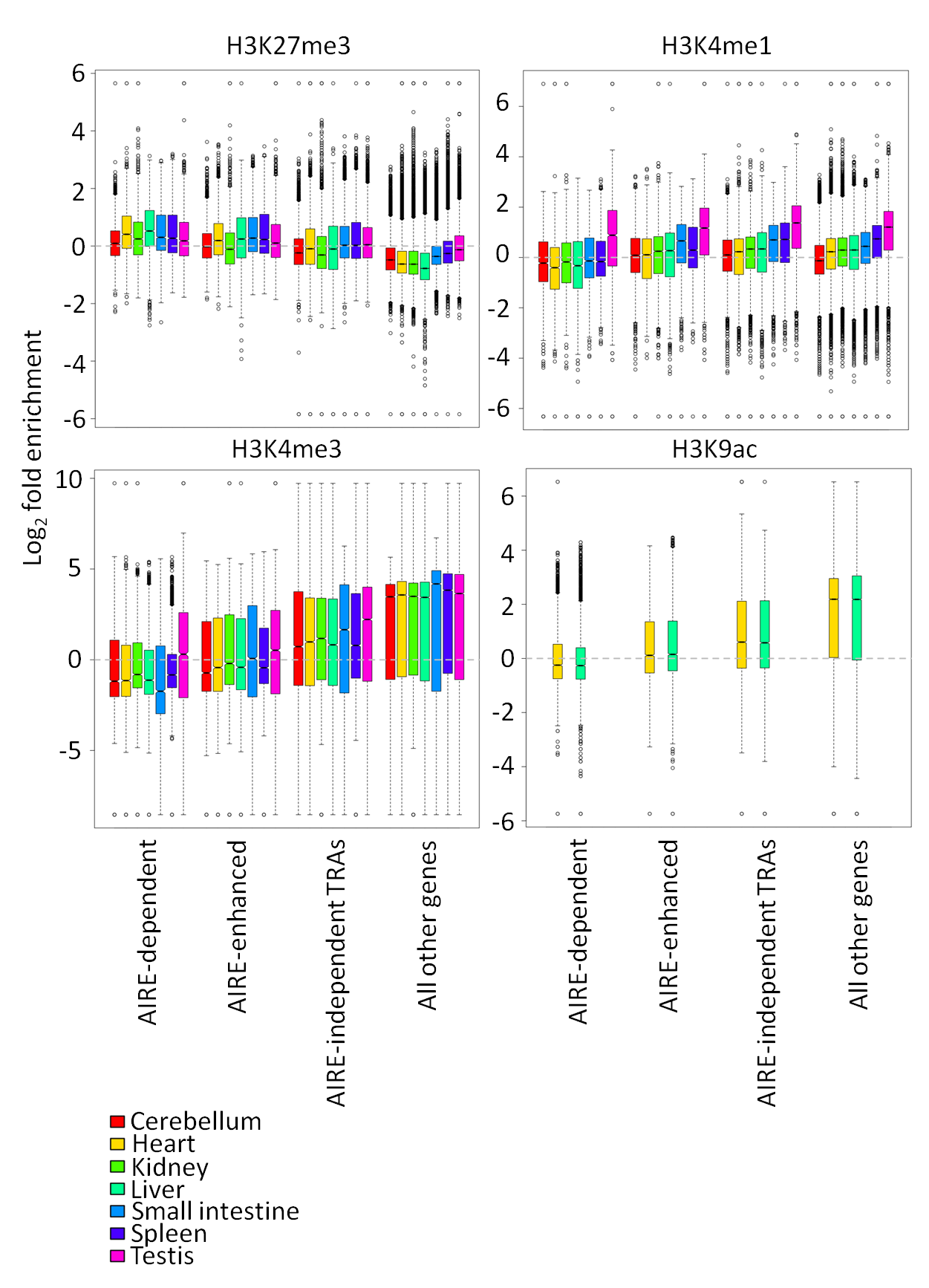

Supplement: Supplementary Figure 4 — Chromatin signals in ENCODE tissues. Boxplots show log2 ChIP/input ratio scaled by library size within 1 kb of the TSS of genes that are AIRE-dependent, AIRE-enhanced, AIRE-independent TRAs or all other genes. Kruskal-Wallis p < 0.0001 for all tissues. [file Image_4.PNG]

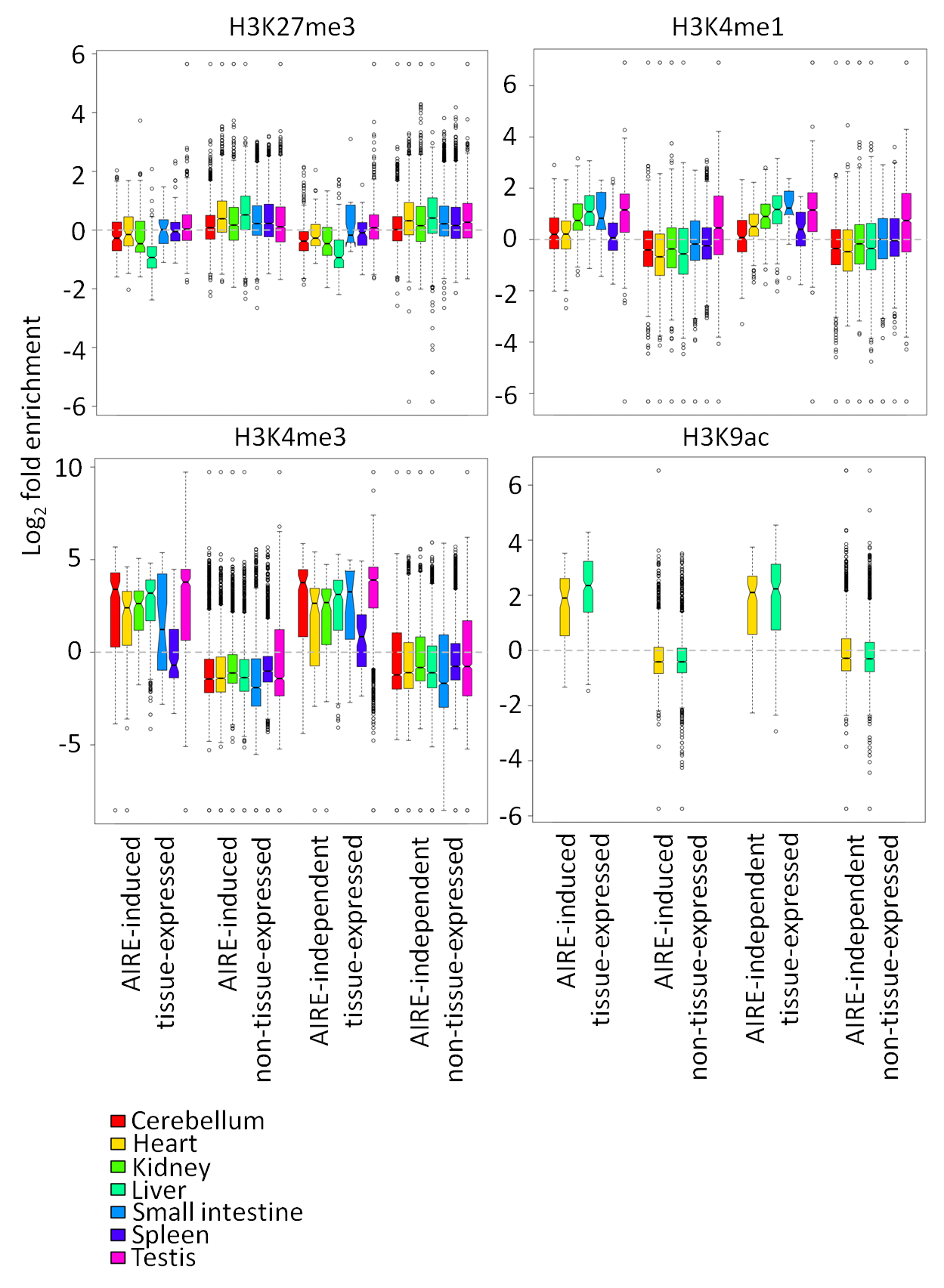

Supplement: Supplementary Figure 5 — Chromatin signals in ENCODE tissues near tissue specific genes. Boxplots show log2 ChIP/input ratio scaled by library size within 1 kb of the TSS of genes with tissue specificity tau ≥ 0.8. Genes are divided into AIRE-induced and AIRE-independent, and additionally for each tissue into genes maximally expressed in each tissue or not maximally expressed in that tissue. Kruskal-Wallis p < 0.05 for all tissues. [file Image_5.PNG]

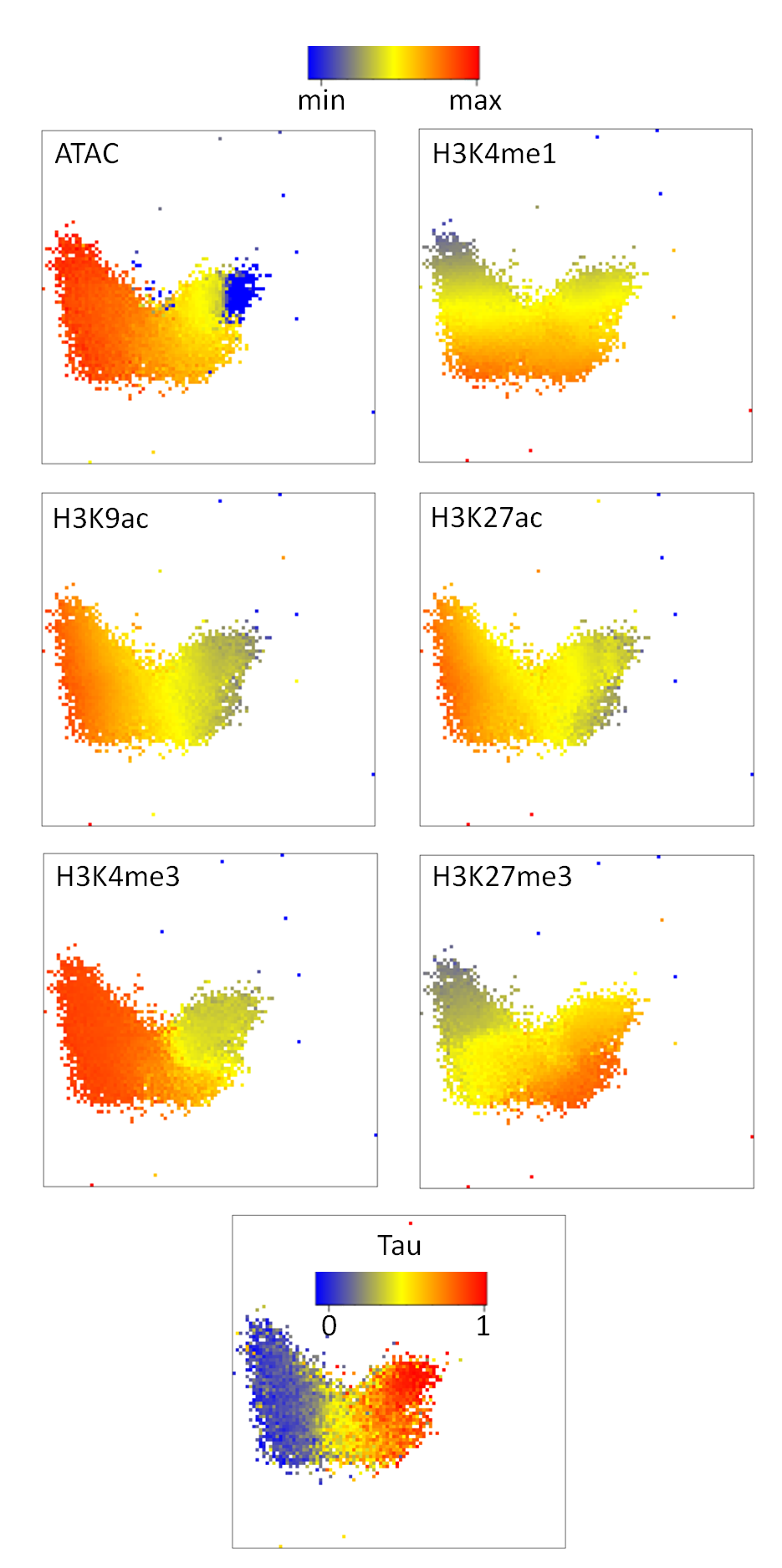

Supplement: Supplementary Figure 6 — Heatmaps of chromatin signals in mTEClo. ATAC-seq signal is expressed as log2 CPM+1. ChIP-seq signal is expressed as log2 ChIP/input ratio scaled by library size. The bottom panel shows tau tissue specificity index. [file Image_6.PNG]

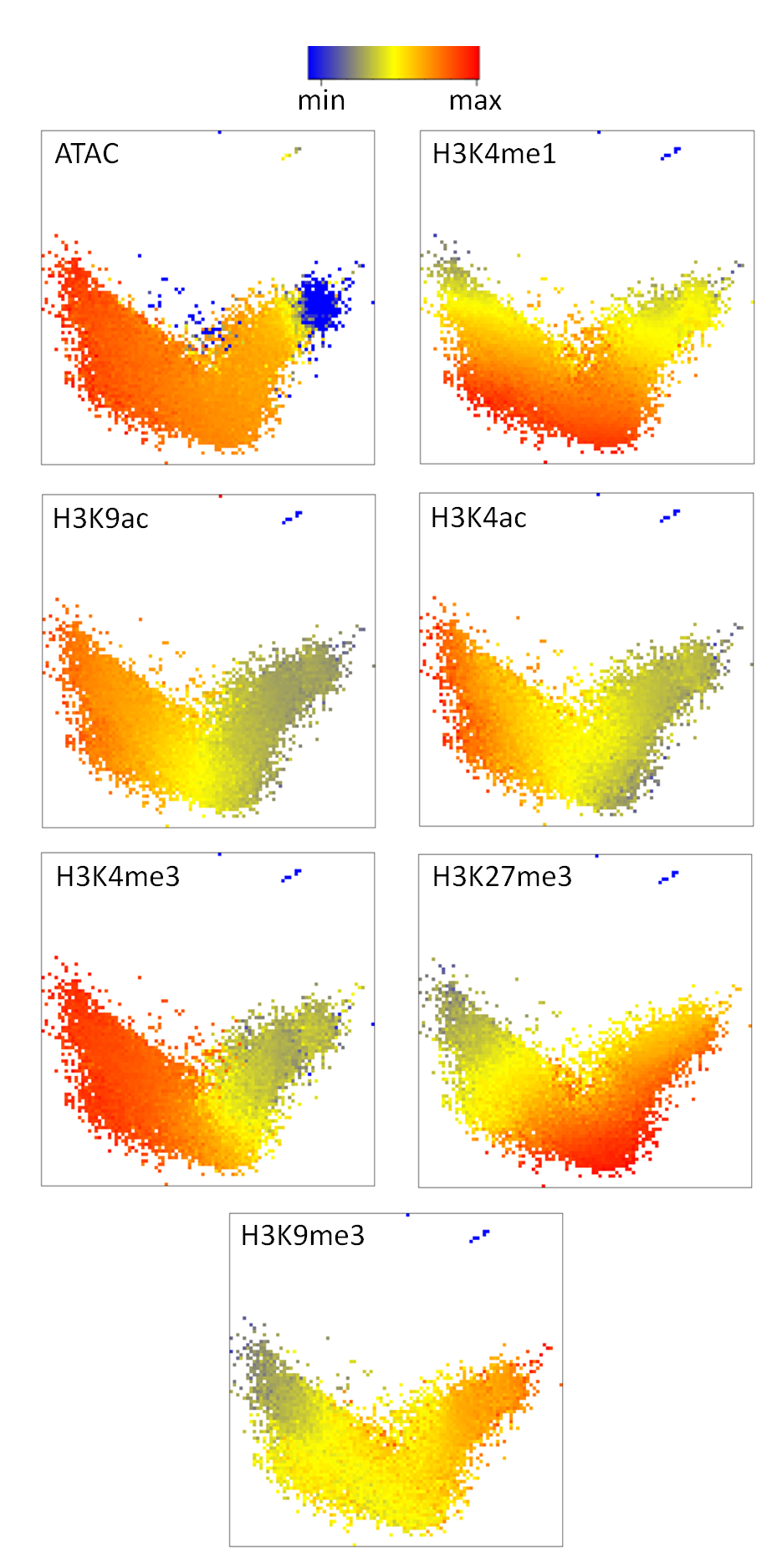

Supplement: Supplementary Figure 7 — Heatmaps of chromatin signals in mTEChi. ATAC-seq signal is expressed as log2 CPM+1. ChIP-seq signal is expressed as log2 ChIP/input ratio scaled by library size. [file Image_7.PNG]

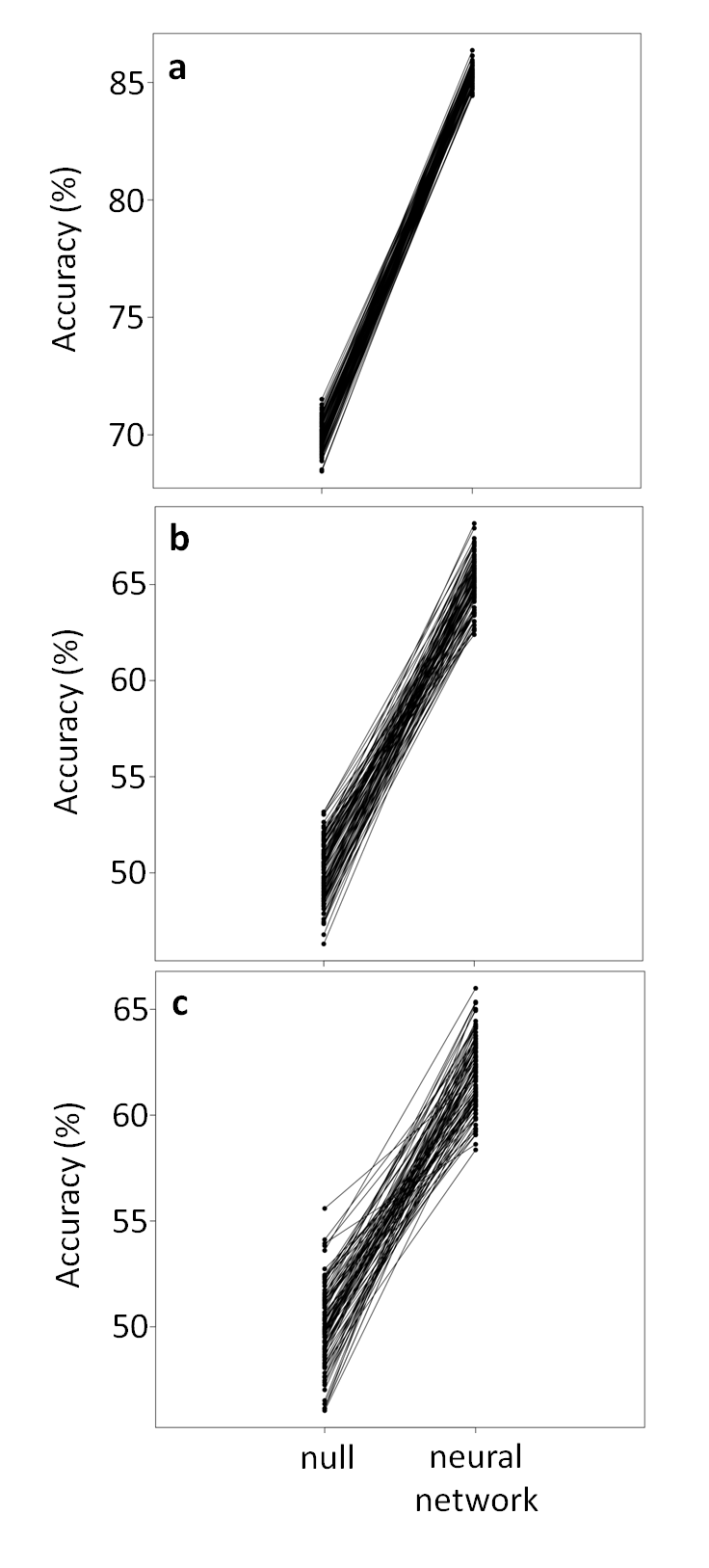

Supplement: Supplementary Figure 8 — Plots of accuracy of neural networks compared with null accuracy. (a) All genes, (b) tissue restricted genes (tissue specificity tau ≥ 0.8), and (c) tissue restricted genes (tissue specificity tau ≥ 0.8) closely matched on proportional expression in single mTEChi. The null accuracy was estimated by randomly sampling the true gene categories for each 100 neural networks. [file Image_8.PNG]

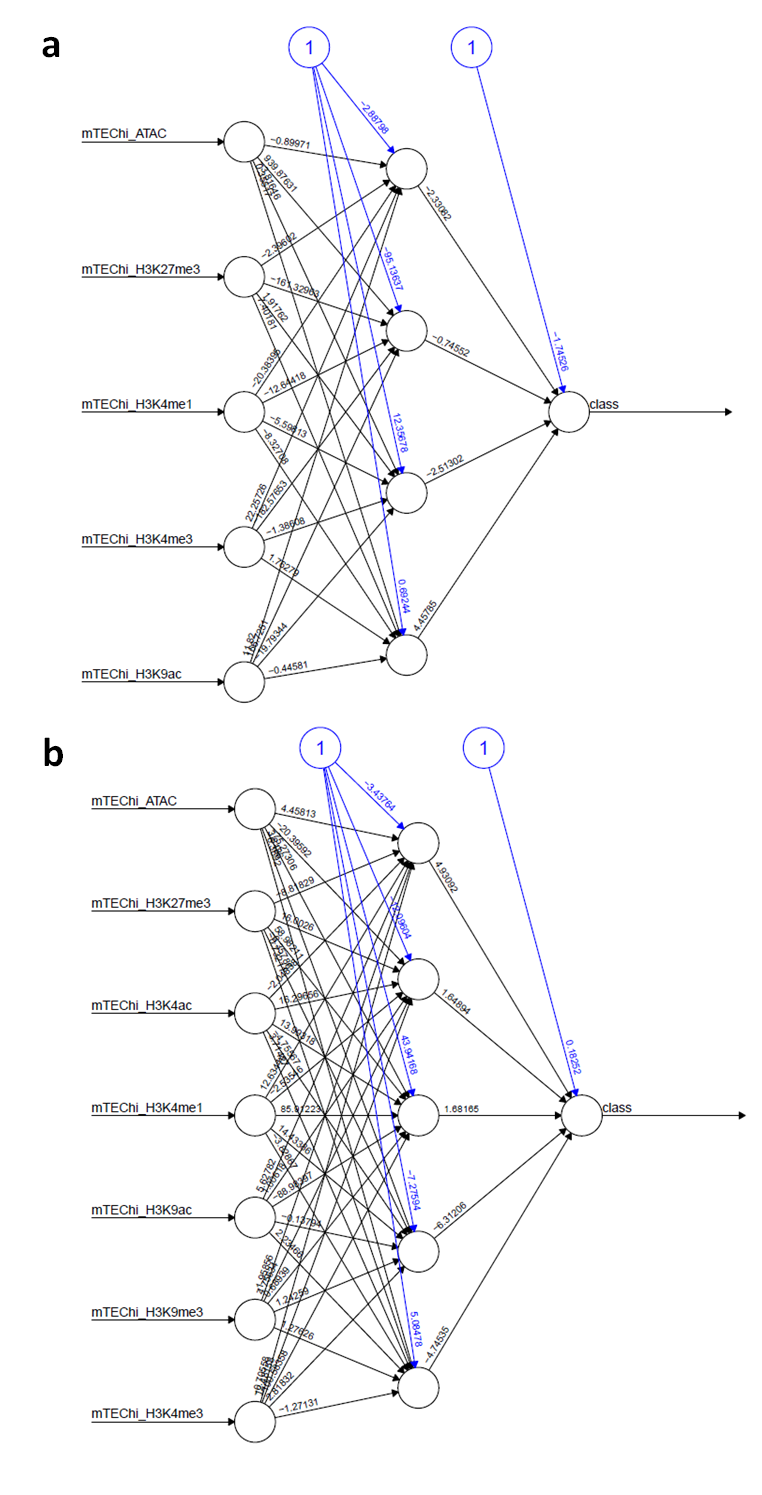

Supplement: Supplementary Figure 9 — Optimum neural networks for AIRE categorization. Plots of the neural networks are shown for the network with the best accuracy for (a) all genes and (b) tissue restricted genes (tau ≥ 0.8). [file Image_9.PNG]

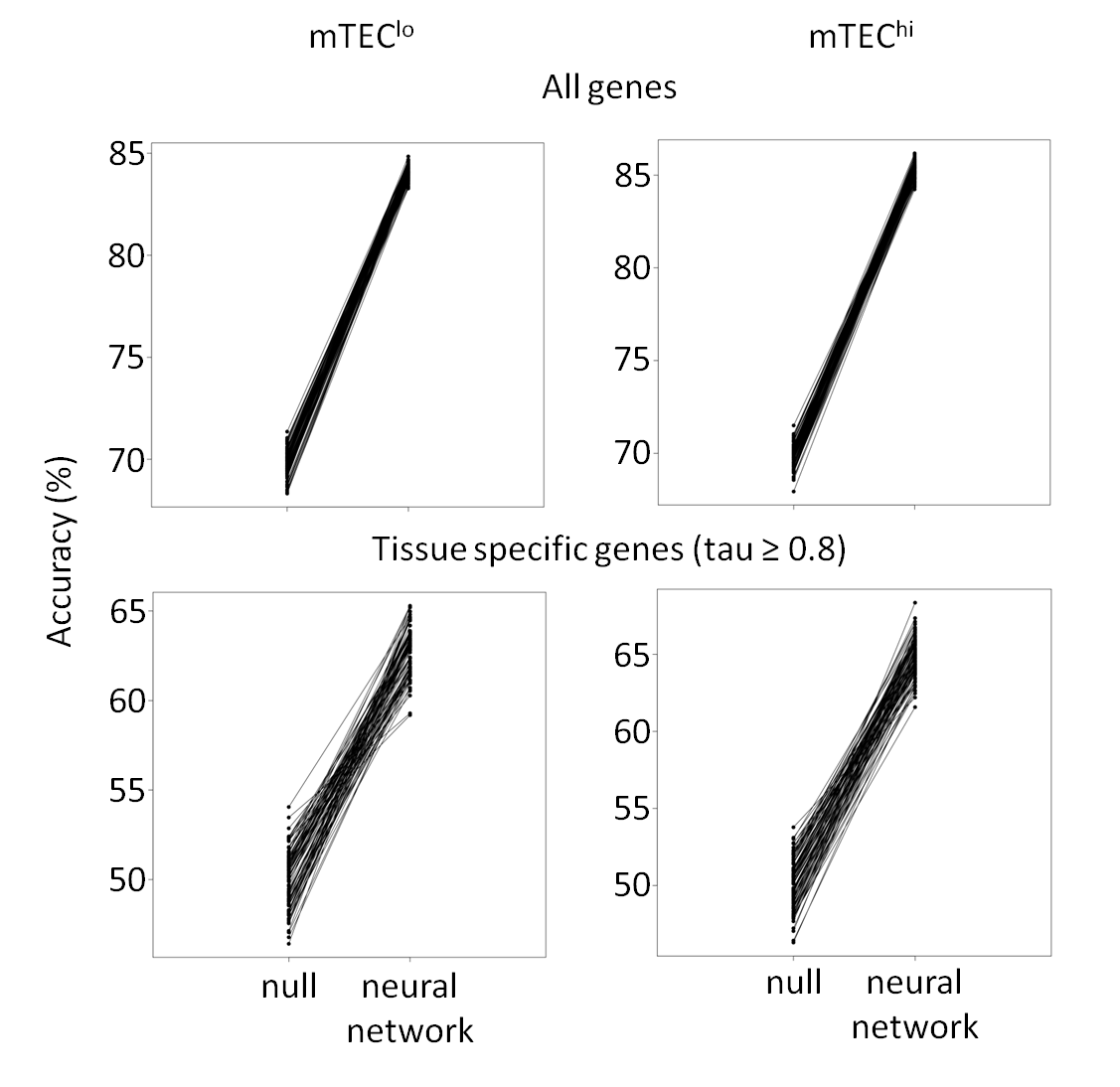

Supplement: Supplementary Figure 10 — Accuracy of neural networks for predicting AIRE status of genes. [file Image_10.PNG]

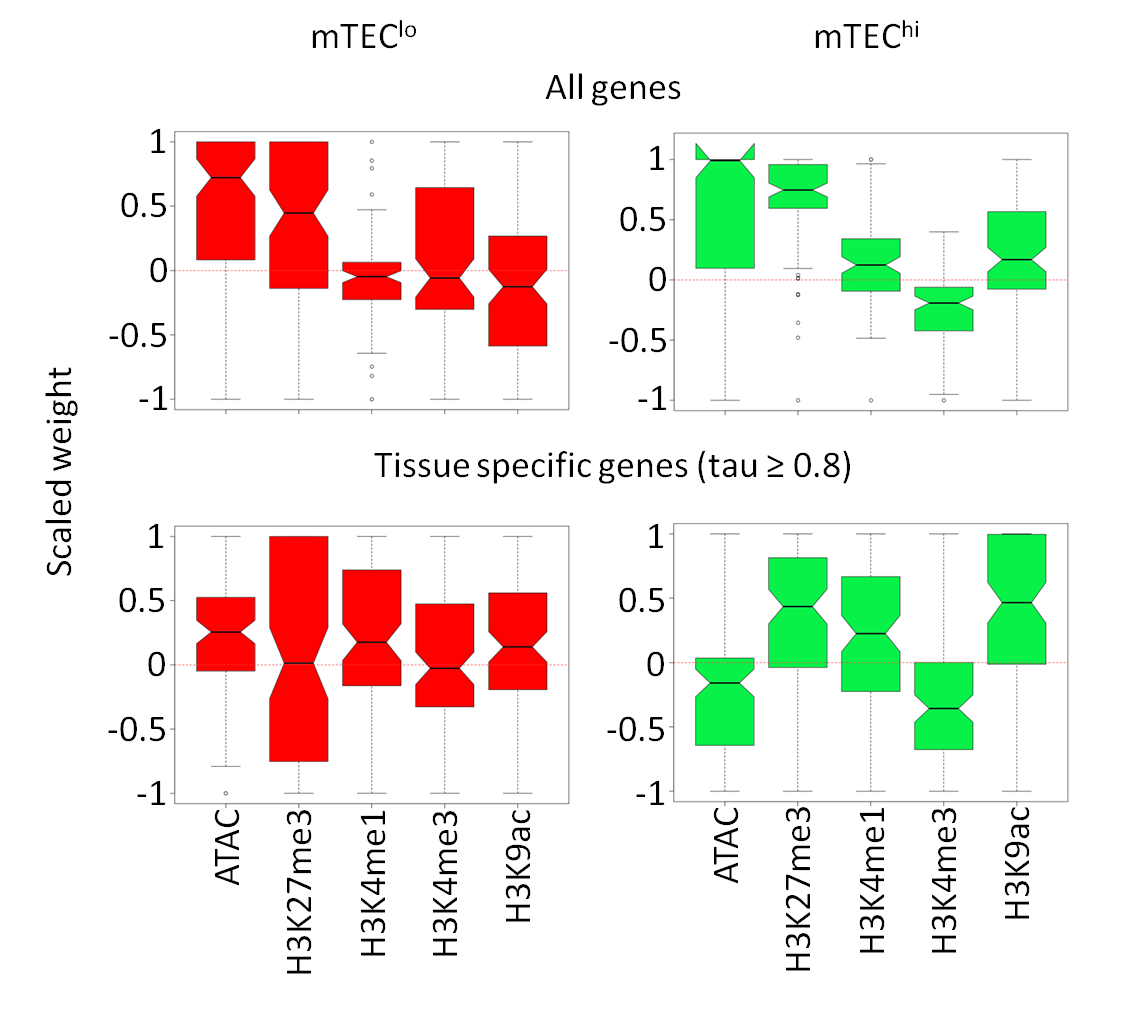

Supplement: Supplementary Figure 11 — Olden weighting of chromatin accessibility and histone modifications in neural networks for predicting AIRE status of genes. [file Image_11.PNG]

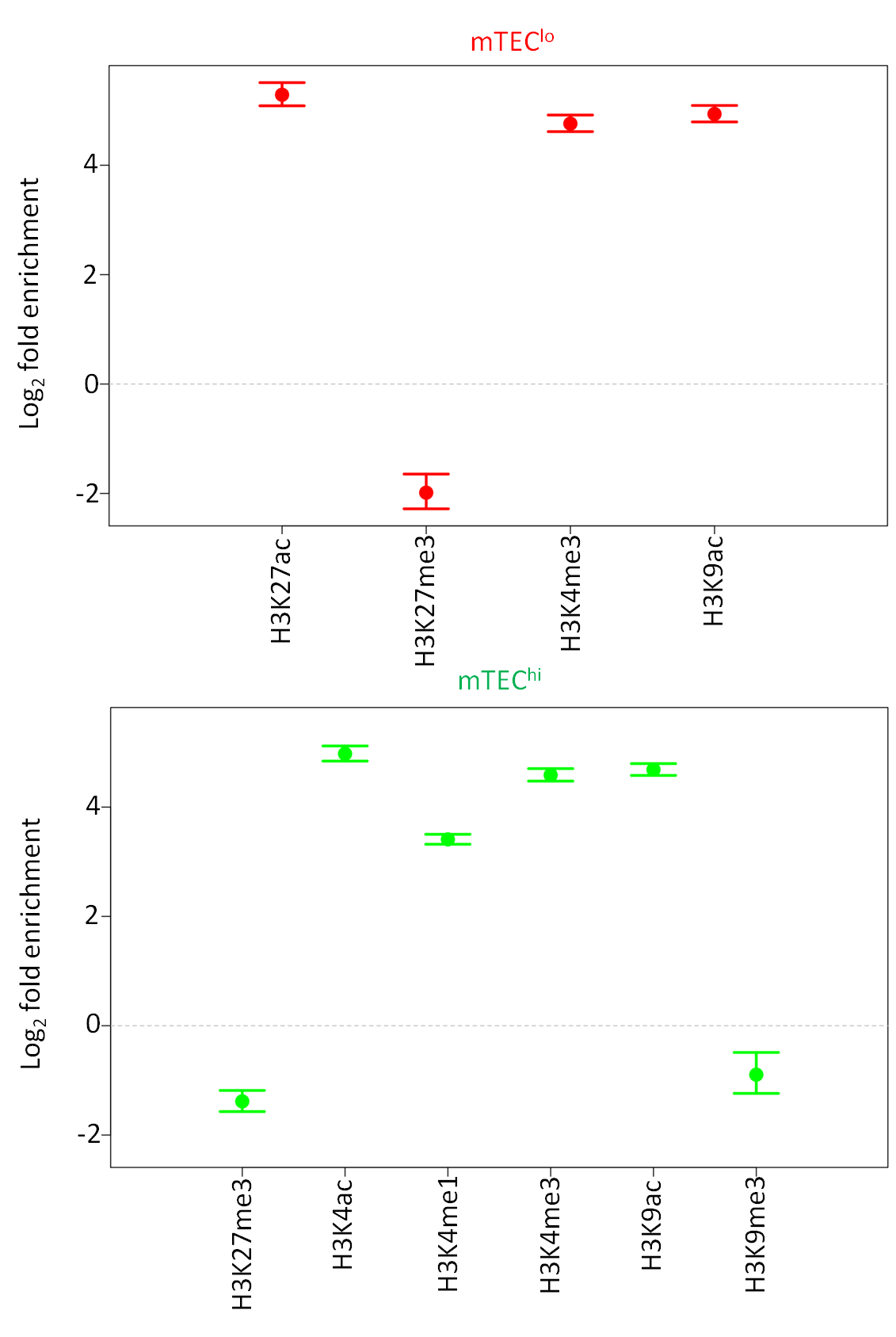

Supplement: Supplementary Figure 12 — Enrichment of chromatin modifications within AIRE binding sites. GAT was used to test enrichment of histone ChIP-seq peaks (IDR < 0.01) in mTEClo (red) and mTEChi (green) within AIRE ChIP-seq peaks (IDR < 0.01) relative to the rest of the mappable genome. Error bars indicate 95% confidence intervals from 10,000 permutations. [file Image_12.PNG]

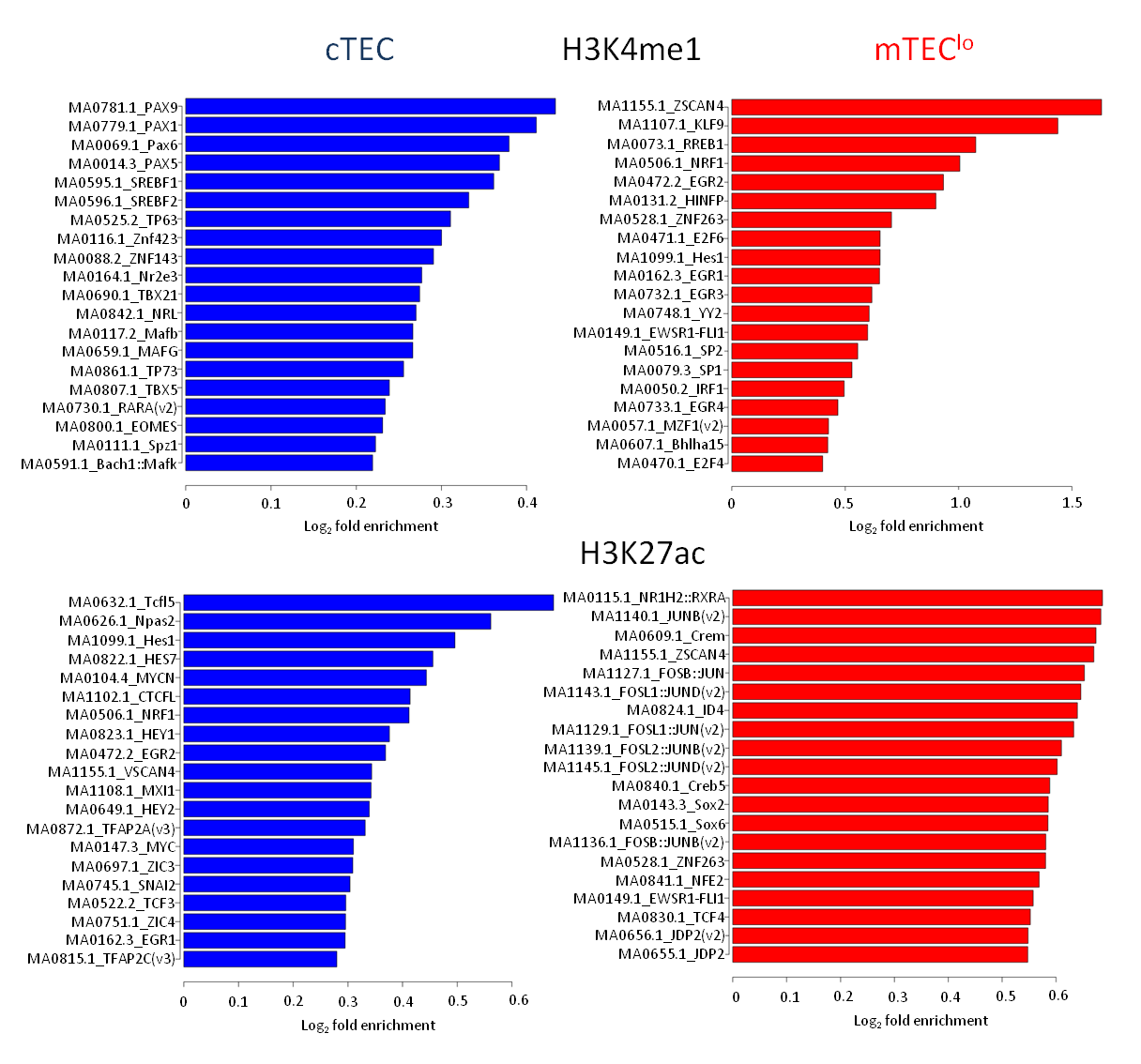

Supplement: Supplementary Figure 13 — Significantly enriched JASPAR motifs in cTEC (blue) or mTEClo (red) for H3K4me1 (top) or H3K27ac (bottom). The top 20 significant motifs (FDR < 0.05) are shown ordered by enrichment. [file Image_13.PNG]

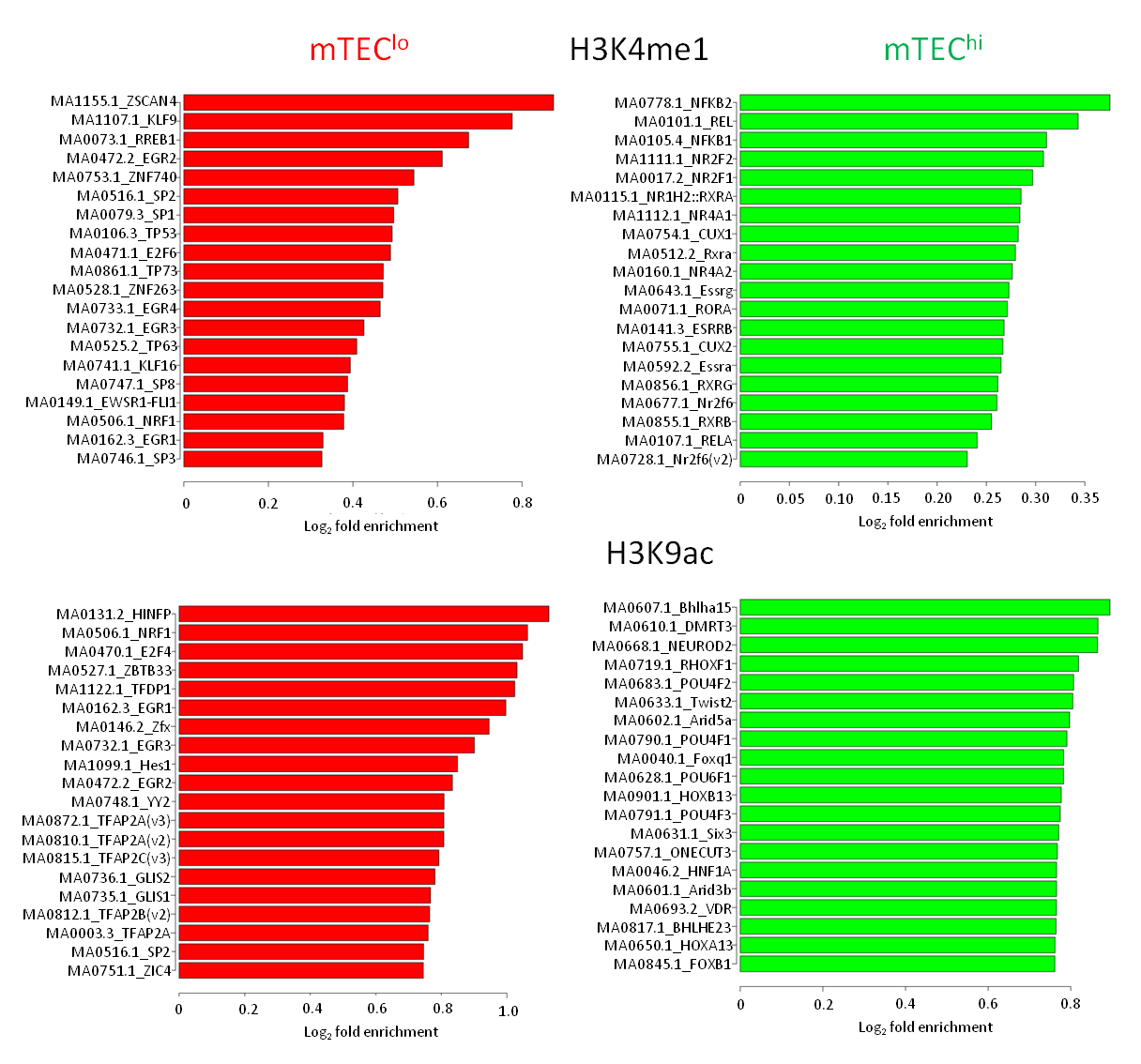

Supplement: Supplementary Figure 14 — Significantly enriched JASPAR motifs in mTEClo (red) or mTEChi (green) for H3K4me1 (top) or H3K9ac (bottom). The top 20 significant motifs (FDR < 0.05) are shown ordered by enrichment. [file Image_14.PNG]

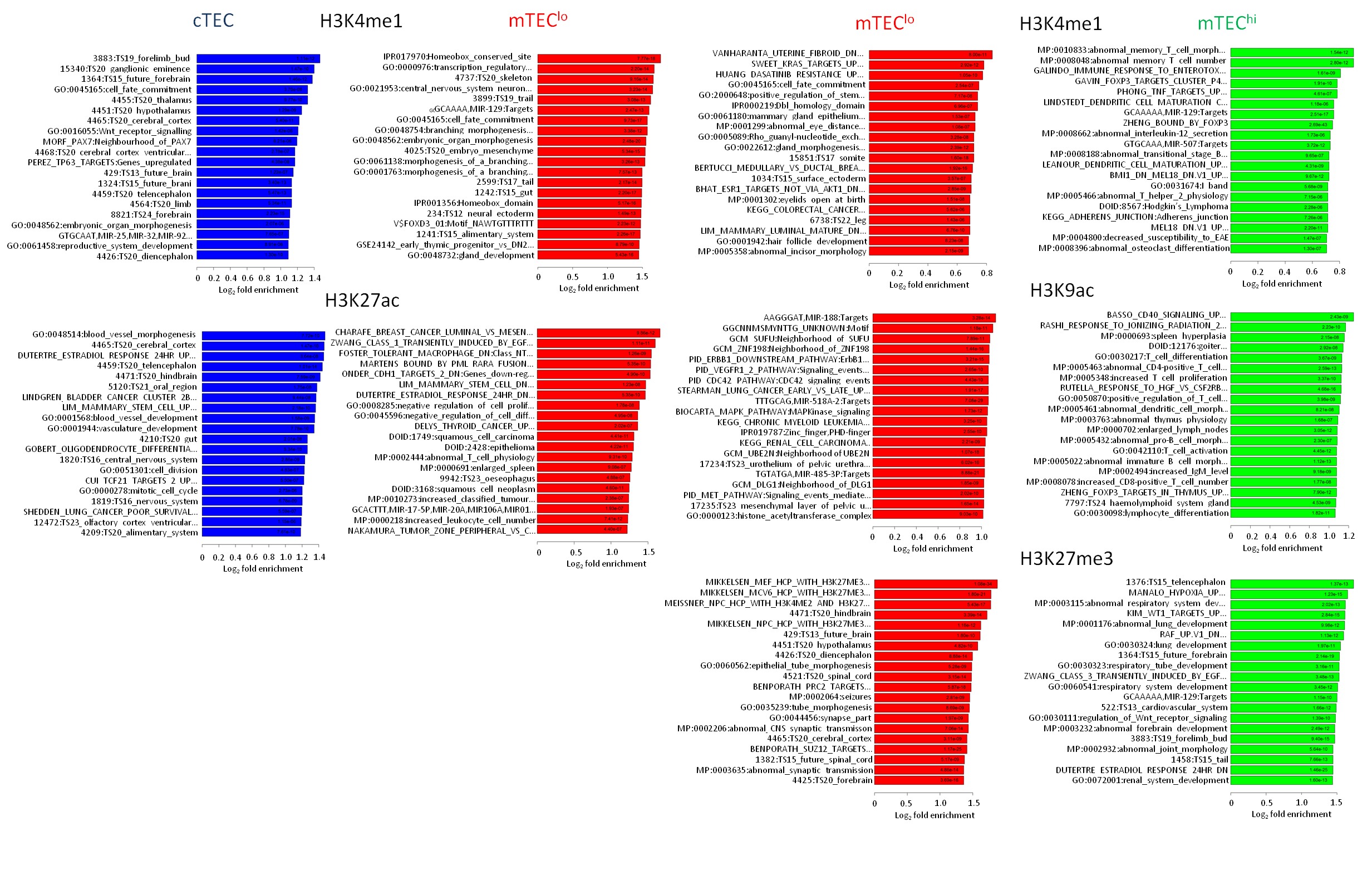

Supplement: Supplementary Figure 15 — (left) Gene ontology enrichment from GREAT on differential peaks in cTEC (blue) or mTEClo (red); (right) Gene ontology enrichment from GREAT on differential peaks in mTEClo (red) or mTEChi (green). The top 20 significant terms (FDR < 0.05) ranked by fold enrichment are shown. Only categories with greater than 50 and fewer than 1,000 genes are shown. [file Image_15.PNG]
